# From drug export to envelope fitness: characterizing the regulatory landscape of selective *Escherichia coli* ABC efflux pumps

**DOI:** 10.3389/fmicb.2026.1889688

**Published:** 2026-09-04

**Authors:** Zhenyue Feng, Meng Wu, Chaohuan Yang, Wei Zheng, Yuxin Wang, Shunxin Wang, Defu Liu

**Affiliations:** 1 College of Agronomy and Life Sciences, Zhaotong University, Zhaotong, China; 2 Yunnan Engineering Research Center of Green Planting and Processing of Gastrodia, Zhaotong University, Zhaotong, China; 3 Institute for Advanced Study on Ecological Agriculture and Biodiversity of the Yunnan-Guizhou Plateau, Zhaotong University, Zhaotong, China; 4 Yunnan Key Laboratory of Smart Villages and Agri-Cultural-Tourism Integration, Zhaotong University, Zhaotong, China

**Keywords:** ABC efflux pump, CpxR, DNA pull-down, drug resistance, *Escherichia coli*, transcriptional regulation, *YbhF*, *YddA*

## Abstract

**Background:**

Antimicrobial resistance remains a top global health threat. While ATP-binding cassette (ABC) efflux pumps contribute to multidrug resistance in Gram-negative bacteria, their transcriptional regulation is far less understood than that of the RND and MFS families.

**Methods:**

We used DNA pull-down followed by LC–MS/MS to identify proteins binding to the promoters of five *E. coli* ABC efflux pump genes (*msbA*, *macB*, *ybhF*, *yddA*, and *yadG*). Candidate regulators were analyzed using bioinformatics, domain scanning, and molecular docking.

**Results:**

Binding patterns differed sharply across the five genes. *MsbA*, *macB*, and *ybhF* pulled down complex interaction networks, while *yddA* and *yadG* bound far fewer regulators. CpxR was among the most highly enriched regulatory proteins, recovered at the *msbA*, *macB*, and *ybhF* promoters with fold changes of 30.57, 57.17, and 17.39, respectively. OmpR and ArcA exhibited significant enrichment at the *ybhF* promoter (FC = 64.77 and 238.28, respectively). Both regulators are implicated in osmotic and redox signaling, suggesting a potential link to *ybhF* transcriptional control under such conditions. LrhA was prominently enriched at the *yddA* promoter (FC = 103.21). This observation raises the possibility of a connection between LrhA and biofilm-related regulatory pathways, which remains to be validated experimentally. Structurally, the *ybhFSR* operon is organized as a split-type system: *ybhF* encodes the nucleotide-binding domains (NBDs), while *ybhS* and *ybhR* encode the transmembrane domains (TMDs). The system likely assembles with YbhG and TolC into a tripartite complex. By contrast, based on structural prediction, *yddA* and *yddB* may form a minimal two-subunit ABC transporter, marked by atypical Walker A (GYSGAGKTT) and ABC signature (LSSGE) motifs.

**Conclusion:**

Our data show that ABC pumps are tightly integrated into a regulatory network governing envelope stress, osmotic adaptation, and biofilm formation—not just drug export. These findings identify candidate regulators whose putative promoter binding requires experimental validation.

## 1 Introduction

Antimicrobial resistance has emerged as a major global public health threat, accompanied by a declining pipeline of novel antimicrobial agents (Darby et al., 2023; Novelli and Bolla, 2024). Recent work has highlighted that antimicrobial resistance is shaped not only by antibiotic exposure but also by ecological and metabolic factors that influence the resistome across microbial communities (Gahlot, 2026). The World Health Organization has classified multidrug-resistant Gram-negative bacteria as high-priority pathogens, since efflux pump overexpression represents a key contributor to clinical antimicrobial tolerance and the horizontal spread of resistance phenotypes (Liu et al., 2024; Athar et al., 2023). Efflux alone, however, does not account for all modes of AMR dissemination. Conjugative transfer and biofilm formation represent additional routes for AMR dissemination beyond efflux activity. Patkowski et al. (2023) showed that F-pili, which are prevalent in Enterobacteriaceae, resist thermal and hydrodynamic stresses in the gut and body fluids. These pili mediate horizontal transfer of IncF multidrug-resistance plasmids and promote biofilm maturation via cell aggregation. Biofilm microenvironments increase bacterial tolerance to antibiotics, and together with conjugation, this accelerates resistance gene spread. Yet most mechanistic studies focus on intracellular efflux through RND, MFS and ABC transporters, while conjugation- and biofilm-driven pathways receive far less attention. We contend that resistance involves a combination of efflux, plasmid transfer and biofilm protection, and that efflux-centered studies offer an incomplete view. Within the efflux systems themselves, RND-type transporters are widely acknowledged as the primary mediators of high-level multidrug resistance in Gram-negative bacteria, while ABC efflux transporters are important secondary contributors to intrinsic and acquired drug tolerance (Orelle et al., 2019). Powered by ATP hydrolysis, ABC transporters mediate antimicrobial extrusion, endogenous substrate translocation, and adaptive responses to extracellular stress. However, their transcriptional regulatory networks remain less well characterized than those of RND and MFS families, even in the model organism *E. coli*. Elevated ABC expression facilitates bacterial survival under antibiotic pressure, and understanding the transcriptional circuits governing these transporters is essential to decipher the molecular basis of clinical resistance and identify potential adjuvant therapeutic targets.

Before examining how these transporters are regulated at the transcriptional level, it is useful to consider the conserved structural features that define their catalytic activity. All functional ABC transporters rely on conserved nucleotide-binding domains (NBDs) to drive substrate translocation, where six canonical sequence motifs collectively constitute a complete ATP catalytic cycle (Rees et al., 2009). The Walker A motif [consensus GXXXXGK(S/T)] employs a central lysine residue to coordinate ATP phosphate groups, with the second conserved glycine maintaining flexible loop conformations required for nucleotide docking (Walker et al., 1982). The Q-loop contains a conserved glutamine that mediates Mg^2+^ chelation and allosteric communication between NBD and transmembrane domain (TMD) subunits (Jones and George, 2004). The ABC signature motif (LSGGQ) forms a bipartite ATP-binding pocket by interacting with the Walker A domain of the opposing NBD monomer (Smith et al., 2002). The Walker B motif contains a hydrophobic stretch terminating in Mg^2+^-chelating aspartate, with an adjacent glutamate serving as the catalytic residue for ATP hydrolysis (Moody et al., 2002). The D-loop mediates conformational rearrangement of NBD dimers after ATP turnover, and the conserved histidine within the H-switch motif stabilizes the hydrolysis transition state (Oldham and Chen, 2011). Comparative functional analysis of divergent ABC proteins should prioritize variation within these catalytic core residues rather than overall sequence homology (Rees et al., 2009).

Transcriptional reprogramming constitutes a major regulatory layer controlling bacterial efflux pump expression, mediated by signaling networks that integrate environmental cues and antimicrobial stress (Weston et al., 2018; De Gaetano et al., 2023). Two-component systems (TCS) represent the primary stress-sensing machinery, consisting of membrane-anchored histidine kinases and cytoplasmic response regulators that activate or repress downstream genes via phosphorylation cascades. Conserved Gram-negative TCS, including CpxAR, EnvZ/OmpR, and PhoPQ, govern outer membrane integrity, antimicrobial peptide tolerance, and drug susceptibility, though direct regulatory links between PhoPQ and ABC efflux pumps lack empirical validation in *E. coli* (Beier and Gross, 2006; De Gaetano et al., 2023; Bisht et al., 2024). Notably, CpxAR also bridges envelope stress and biofilm regulatory circuits, as validated in *Yersinia* where activated CpxR modulates biofilm formation (Gahlot et al., 2022). Beyond TCS, nucleoid-associated H-NS acts as a global transcriptional silencer by binding AT-rich promoter sequences to repress horizontally acquired resistance and efflux genes. Upon environmental stimulation, competing transcriptional factors displace H-NS to de-repress target loci, a silencing–de-repression axis hypothesized to modulate ABC pump expression (Liu and Wang, 2025). Additional pleiotropic transcription factors, including MarA, ArcA, and LrhA, coordinate antimicrobial tolerance, motility, and biofilm biogenesis, indicating that ABC transporters are part of integrated stress response networks (Weston et al., 2018). Our previous work showed that the *E. coli* YbhFSR ABC transporter confers tetracycline resistance and mediates Na^+^/Li^+^ antiport, yet its upstream transcriptional regulators and regulatory logic remain uncharacterized (Feng et al., 2020).

In this study, five representative *E. coli* ABC transporter loci were selected to establish a comparative screening framework, encompassing both well-characterized canonical pumps and poorly annotated efflux systems with distinct genomic architectures. Two reference genes, *msbA* and *macB*, encode core subunits of established ABC machineries: MsbA mediates inner-membrane lipopolysaccharide translocation to sustain outer membrane homeostasis, whereas MacB assembles with MacA-TolC to export macrolide antibiotics. Despite their well-characterized transport functions, the transcriptional cascades governing their promoter regions remain largely uncharacterized, making them suitable reference targets for comparative regulatory profiling via DNA pull-down. The remaining three loci encode less characterized ABC systems with distinct operon structures. Prior functional assays verified YbhFSR as a tetracycline efflux and cation antiport system; the *ybhF* gene encodes two NBDs, while *ybhS* and *ybhR* produce TMD subunits, forming a split polycistronic operon that assembles with periplasmic YbhG to generate a four-component efflux complex. Biochemical and MIC testing confirmed YddA as an ATP-dependent multidrug efflux protein carrying atypical Walker A and ABC signature motifs, with *yddA*-*yddB* arranged as a compact bicistronic unit. A companion study (Feng et al., 2020) has shown that YadGH heterodimers mediate antimicrobial extrusion. The divergent structural and functional features of these five ABC transporters collectively provide a valuable basis for comparing conserved and transporter-specific transcriptional regulatory patterns.

To date, most published investigations of ABC efflux regulation focus on single transporters or isolated transcription factors, with very few parallel comparative analyses covering multiple ABC family members. Such single-gene experimental designs limit systematic insight into shared co-regulatory networks driving adaptive bacterial resistance. DNA pull-down coupled with LC–MS/MS proteomics provides a high-throughput approach to capture native promoter-interacting proteins and map transcriptional regulatory networks. In this work, we combined well-defined reference systems (*msbA*, *macB*) and understudied ABC operons (*ybhFSR*, *yddA*-*yddB*, *yadGH*) to conduct parallel pull-down screening. This multi-target design allowed us to assess the reliability of our proteomic workflow while uncovering candidate regulatory factors, providing a global overview of ABC transcriptional networks under basal drug-free conditions, and establishing a foundation for follow-up functional verification of predicted protein–promoter interactions as well as the development of efflux-targeted anti-resistance strategies.

## 2 Materials and methods

### 2.1 Bacterial culture and reagent preparation

*E. coli* K-12 strain MG1655 (ATCC 700926) was cultured in LB medium at 37 °C with shaking at 200 rpm to an OD₆₀₀ of 0.6. Genomic DNA was isolated and served as the template for PCR amplification of promoter probes. Primers targeting the promoter regions of *yadG*, *ybhF*, *msbA*, *macB*, and *yddA* were synthesized with 5′ biotin modifications by Sangon Biotech (Shanghai, China). Biotinylated probes were amplified using KOD-Plus-Neo high-fidelity DNA polymerase (Toyobo, KOD-201) and purified with the Universal DNA Purification Kit (Tiangen, DP214). Streptavidin-coupled magnetic beads (Invitrogen, 11205D) were utilized for subsequent DNA pull-down enrichment. Cells were lysed in RIPA buffer (50 mM Tris–HCl, pH 7.5; 150 mM NaCl; 1% NP-40; 0.5% sodium deoxycholate; 0.1% SDS) supplemented with 1 mM PMSF and protease–phosphatase inhibitor cocktail (Sigma, P0044).

### 2.2 DNA pull-down assay

All DNA pull-down procedures and SDS-PAGE validation were carried out in our laboratory. Eluted protein samples were sent to BioGenes GmbH for LC–MS/MS detection, while all downstream proteomic bioinformatic analyses and data interpretation were independently conducted by our research team.

#### 2.2.1 Preparation of biotin-labeled DNA probes

Biotinylated promoter fragments of *yadG*, *ybhF*, *msbA*, *macB*, and *yddA* were amplified from *E. coli* MG1655 genomic DNA. As summarized in Table 1, the reverse primer within each primer pair carried a 5′ biotin tag. Equimolar unbiotinylated promoter amplicons were separately generated as cold competitive controls to distinguish specific protein-DNA binding from non-specific electrostatic interactions.

**Table 1:** Sequences of primers used in this study.

Primer name	Sequence (5′ → 3′)	Purpose/Notes
PB1-25010181-YbhF-biotin F	TCCAGCACCGCAGGGATCG	Biotin-labeled forward primer for amplification of the *ybhF* promoter probe.
PB1-25010181-YbhF R	GTCCTGCCTCGTCACCGAATTG	Reverse primer for amplification of the *ybhF* promoter probe.
PB1-25010181-YbhF-cold F	TCCAGCACCGCAGGGATCG	Non-biotinylated forward primer for preparation of the unlabeled probe (negative control).
PB1-25010181-MsbA-biotin F	TACAAGGTCGACGTATATTCCCTG	Biotin-labeled forward primer for amplification of the *msbA* promoter probe.
PB1-25010181-MsbA R	TCAAAAAACCAGCATTTGTTGAAA	Reverse primer for amplification of the *msbA* promoter probe.
PB1-25010181-MsbA-cold F	TACAAGGTCGACGTATATTCCCTG	negative control.
PB1-25010181-MacB-biotin F	CAGGCTGCGGTAAATATGCGTTT	Biotin-labeled forward primer for amplification of the *macB* promoter probe.
PB1-25010181-MacB R	TGTGCAGCTCCTGGTTTGG	Reverse primer for amplification of the *macB* promoter probe.
PB1-25010181-MacB-cold F	CAGGCTGCGGTAAATATGCGTTT	negative control.
PB1-25010181-YddA-biotin F	GCAATGGATTTCTACCCGA	Biotin-labeled forward primer for amplification of the *yddA* promoter probe.
PB1-25010181-YddA R	TAGCGAAAATTGATTGTGCCA	Reverse primer for amplification of the *yddA* promoter probe.
PB1-25010181-YddA-cold F	GCAATGGATTTCTACCCGA	negative control.
PB1-25010181-YadG-biotin F	GGTGCCGTTGATCTCCTGC	Biotin-labeled forward primer for amplification of the *yadG* promoter probe.
PB1-25010181-YadG R	AAATTTTTACTTACCTTACGTTCTTAC	Reverse primer for amplification of the *yadG* promoter probe.
PB1-25010181-YadG-cold F	GGTGCCGTTGATCTCCTGC	negative control.

The PCR thermal profile consisted of an initial 2 min denaturation step at 94 °C, followed by 35 cycles: 15 s at 94 °C for denaturation, 30 s annealing at 55–58 °C (adjusted to 5 °C below the Tm of each target fragment), and extension at 68 °C (1 min per kilobase). A final 10 min extension at 68 °C was performed to complete amplification. PCR products were separated on 1% agarose gels; target DNA bands were excised and purified using the Universal DNA Purification Kit (Tiangen, DP214). Purified biotinylated probes were stored at −20 °C prior to pull-down incubation.

#### 2.2.2 Total protein extraction

Single colonies of *E. coli* K-12 were inoculated into LB liquid medium and cultured at 37 °C with shaking at 200 rpm until reaching an OD₆₀₀ of 0.6. A 50 mL culture aliquot was centrifuged at 8,000 × g for 10 min at 4 °C. Cell pellets were washed twice with pre-chilled PBS and resuspended in 1 mL RIPA lysis buffer supplemented with 1 mM PMSF, 1 mM DTT, as well as protease and phosphatase inhibitor cocktails. The suspension was incubated on ice for 20 min with brief vortexing every 5 min, followed by sonication on ice (20% amplitude, 3 s pulse on/3 s pulse off) for 5 min. Lysates were centrifuged at 12,000 × g for 10 min at 4 °C, and the resulting supernatants were collected as total protein extracts. Protein concentrations were quantified via BCA assay; extracts were aliquoted and stored at −80 °C for subsequent use.

#### 2.2.3 Capture and elution of DNA-protein complexes

For biotinylated probe immobilization, streptavidin magnetic beads (30 μL) were washed three times with 500 μL Nucleic Dilution Buffer, then incubated with 100–300 pmol biotinylated DNA probe. Two distinct negative control groups were set up: (i) cold probe control containing equimolar unbiotinylated promoter fragments; (ii) blank bead control without any DNA template. The reaction system was adjusted to a total volume of 500 μL using Nucleic Dilution Buffer and rotated at room temperature for 2 h.

For protein incubation, beads were captured with a magnetic rack and washed three times with Nucleic Dilution Buffer. Washed beads were resuspended in 450 μL total protein extract, and the volume was brought to 1 mL with Protein Dilution Buffer. The mixture was incubated overnight at 4 °C under constant rotation. A 100 μL portion of whole protein lysate was retained as the Input control, mixed with SDS-PAGE loading buffer and stored at −20 °C.

For washing and elution, beads were washed five times with 1 mL Protein Dilution Buffer each. Bound proteins were eluted by heating at 95 °C for 8–10 min in 100 μL Elution Buffer, and the recovered supernatant was collected as pull-down product.

For sample preparation, eluted samples (100 μL) were supplemented with 20 μL 6 × SDS-PAGE loading buffer, denatured at 95 °C for 5 min, and subjected to silver staining or Western blot detection.

#### 2.2.4 Experimental replicates and quality control

All DNA pull-down workflows were performed with two independent biological replicates, including separate bacterial cultivation, protein extraction and full incubation-elution procedures. Only proteins reproducibly detected in both replicates were retained for downstream proteomic analysis. Uniform filtering standards were applied to LC–MS/MS datasets, consistent with the statistical pipeline described in Section 2.3: (i) ≥ 2 unique peptides assigned to each protein; (ii) protein-level FDR < 1%; (iii) fold change ≥ 2 relative to pooled signal intensities of the two negative control groups. The reproducibility of protein identification across biological replicates is visualized in Supplementary Figure S5D.

### 2.3 Mass spectrometry data acquisition and differential enrichment analysis

Protein bands were excised from SDS-PAGE gels and processed via in-gel digestion prior to LC–MS/MS identification. Raw MS spectra were searched against the *E. coli* K-12 MG1655 reference proteome (UniProt database) using MaxQuant v1.6.17.0. Trypsin was set as the digestion protease, permitting a maximum of two missed cleavage sites. Cysteine carbamidomethylation was set as a fixed modification, while methionine oxidation was specified as a variable modification. Mass tolerance thresholds were set to 20 ppm for MS1 precursors and 0.5 Da for MS2 fragment ions. Peptide-level confidence was required to exceed 99%, and the protein identification FDR was maintained below 1%. Protein quantification was performed using the built-in iBAQ algorithm within MaxQuant.

For differential enrichment statistics, raw iBAQ intensities from two biological replicates were log₂-transformed. Protein signals absent in either the pull-down group or matched negative control were imputed from the minimal background intensity across all samples. Log₂ (iBAQ) values were compared between each pull-down sample and its corresponding control using two-tailed unpaired Student’s *t*-tests, followed by Benjamini–Hochberg correction to generate FDR-adjusted *p*-values. Dual statistical thresholds were applied: proteins with log₂ (fold change) ≥ 1 (raw FC ≥ 2) and FDR-adjusted *p* < 0.05 were classified as significantly enriched, whereas those meeting log₂ (fold change) ≤ −1 and FDR < 0.05 were classified as significantly depleted. Proteins falling between these thresholds were categorized as non-significant. Volcano plots were constructed in R using the ggplot2 package, with dashed threshold lines annotated at log₂FC = 1 and FDR = 0.05.

### 2.4 Functional annotation and enrichment analysis

Proteins satisfying the dual screening criteria outlined in section 2.3 were defined as differentially enriched candidates, exhibiting markedly higher iBAQ signals in pull-down fractions relative to all negative controls. Functional characterization was performed using UniProt, the COG (Clusters of Orthologous Groups) database, and DAVID v6.8 to assign Gene Ontology (GO; biological process, molecular function, cellular component) and KEGG pathway terms, with enrichment significance set at *p* < 0.05. Protein–protein interaction (PPI) networks were constructed using the STRING database (v12.0) with *E. coli* K-12 as the reference organism; interactions with a combined confidence score ≥ 0.7 were retained as high-confidence and visualized via the STRING web interface.

### 2.5 Conserved domain and multiple sequence alignment analysis

Conserved domain screening was conducted using the NCBI CDD (v3.23) and SMART (v9.0) databases. CDD search parameters were set as follows: *E*-value ≤ 0.01, bit score ≥ 50, and query coverage ≥ 70%. SMART analyses were run under default settings with a confidence cutoff ≥ 0.5. Domain boundaries were annotated according to published structural coordinate data.

Five *E. coli* ABC transporters (MsbA, MacB, YbhF, YddA, YadG) were analyzed. The *S. aureus* Sav1866 exporter was used as the structural reference, given its complete high-resolution crystal structure and archetypal nucleotide-binding domain (NBD) architecture representative of type I ABC exporters (Dawson and Locher, 2006). Six core NBD catalytic motifs—Walker A, Q-loop, ABC signature, Walker B, D-loop, and H-motif—were mapped based on canonical structural literature (Walker et al., 1982; Smith et al., 2002; Jones and George, 2004). Motifs were classified as canonical when all key catalytic residues were conserved: the lysine and second glycine of Walker A; the diglycine core and terminal glutamine within the ABC signature motif; the aspartate–glutamate catalytic dyad of Walker B; the conserved glutamine of the Q-loop; and the conserved histidine of the H-motif. Motifs harboring substitutions at these critical catalytic residues were categorized as divergent. Multiple sequence alignments were generated with Clustal Omega and visualized in Jalview and WebLogo.

### 2.6 Homology modeling and protein–protein docking

Homology models of YbhF, YbhG, and TolC were constructed, yielding predicted confidence scores of 92.28, 89.97, and 88.91%, respectively. These models served as the basis for subsequent protein–protein docking simulations. Putative binding interfaces were analyzed using PDBePISA to characterize hydrogen bonds, salt bridges, and hydrophobic contacts; interacting residue pairs and interatomic distances were annotated for each predicted complex. Importantly, all interaction interfaces described herein represent computational predictions that await biochemical validation.

### 2.7 Protein–protein interaction network prediction

PPI relationships among YbhG, YbhF, TolC were predicted using the STRING database (v12.0) with *E. coli* K-12 as the background organism. Only interactions with a combined confidence score ≥ 0.7 were considered high-confidence and visualized via the STRING web interface.

## 3 Results

### 3.1 Probe validation and establishment of the DNA pull-down system

To establish a DNA pull-down assay for identifying candidate transcriptional regulators of ABC efflux pumps, specific biotin-labeled probes were designed to target the promoter regions of five target genes: *msbA, macB, ybhF, yddA, and yadG*. Regulatory fragments spanning ~2,000 bp upstream of each translation start site were amplified using KOD-Plus-Neo high-fidelity DNA polymerase and biotinylated primers.

**Figure 1:**
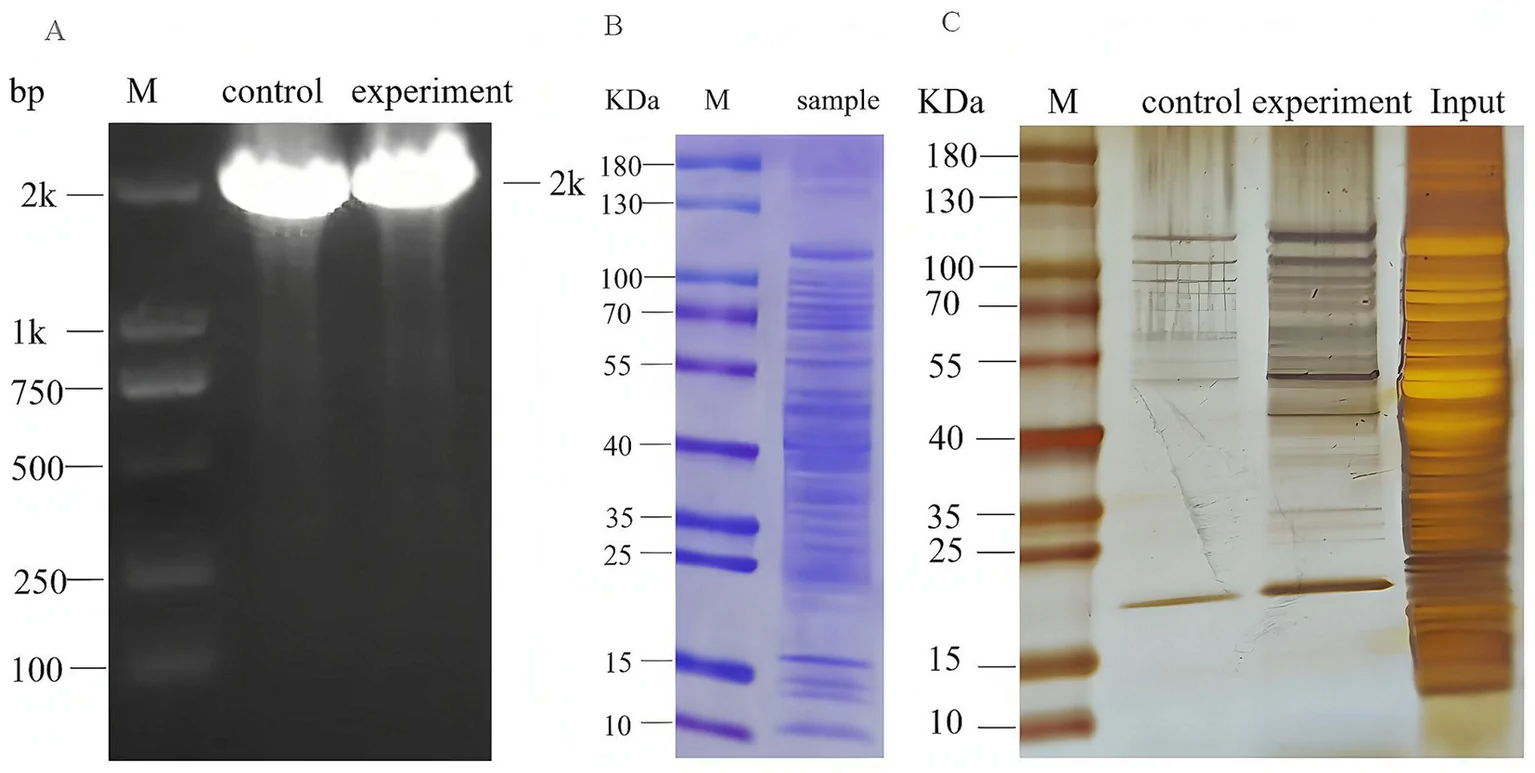
Agarose gel validation of biotinylated promoter fragments and silver-stained SDS-PAGE visualization of protein eluates from DNA pull-down assays. **(A)** Agarose gel electrophoresis to verify the biotinylated msbA promoter fragment (predicted fragment length: 2,000 bp). **(B)** Representative silver-stained SDS-PAGE of pull-down elution fractions. All eluates were normalized via BCA quantification before electrophoresis to achieve equal protein loading across groups. Lanes from left to right: M, protein molecular weight marker; Control, pooled eluates from two negative controls (unbiotinylated competitor probe and bead-only blank); Experiment, proteins captured with biotinylated msbA promoter probe; Input, total soluble whole-cell lysate. Bands ranging from 25 to 130 kDa are observed in the experimental lane; low-molecular-weight bands appear in the control lane. **(C)** Silver-stained SDS-PAGE of whole-cell input lysate, showing band intensity following normalized protein loading.

All five biotinylated PCR products yielded single, discrete bands at the expected molecular weight without visible non-specific amplicons (Figure 1A). Only a representative gel for the *msbA* probe is presented in the main figure; full agarose electrophoresis data for *macB, ybhF, yddA, and yadG* are provided in Supplementary Figures S1–S4, confirming high PCR specificity across all constructs.

Total protein extracts were prepared from *E. coli* in RIPA lysis buffer supplemented with protease and phosphatase inhibitor cocktails. All protein samples were normalized via BCA assay to ensure equivalent loading across the experimental group, Input control, and two distinct negative control groups (Figure 1B). Silver staining showed faint low-molecular-weight background bands in negative control lanes, whereas specific protein bands ranging from 25 to 130 kDa were enriched in experimental pull-down samples (Figures 1B,C).

Collectively, these gel-based quality control data confirm the reliability of our DNA pull-down assay for capturing proteins associating with the promoter sequences of these five ABC efflux pumps.

### 3.2 Statistical analysis and volcano plot visualization of differentially enriched proteins

#### 3.2.1 DNA pull-down and binding protein statistics

To identify candidate proteins associated with the promoters of five *E. coli* ABC efflux pumps (*msbA, macB, ybhF, yddA, and yadG*), DNA pull-down coupled with LC–MS/MS analysis was performed. All pull-down assays were conducted in two independent biological replicates, with >85% overlap in identified protein hits for each promoter, indicating robust experimental reproducibility.

**Figure 2:**
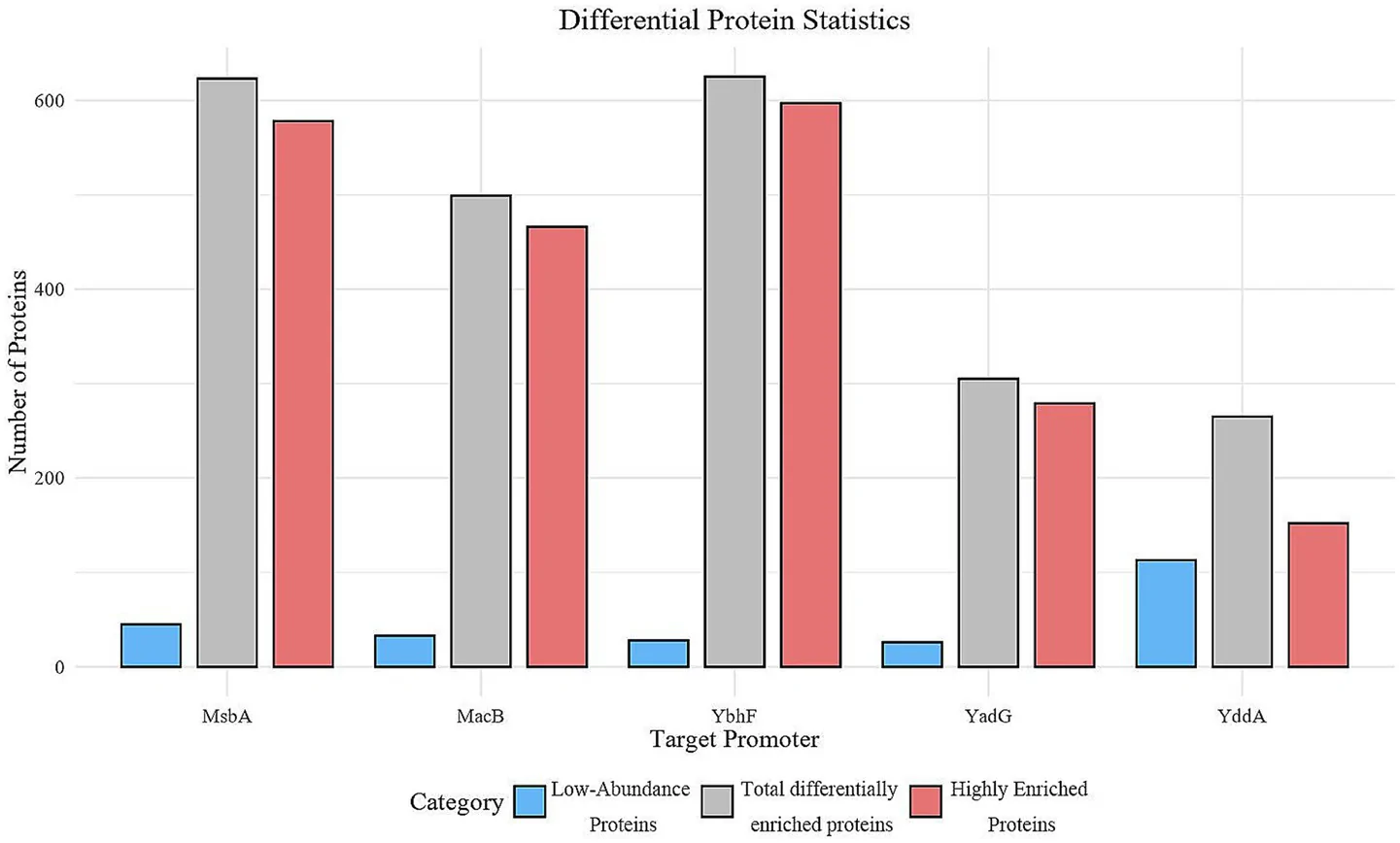
Bar chart quantifying differentially enriched proteins captured by DNA pull-down assays targeting the promoters of five *E. coli* ABC efflux pump genes (*msbA*, *macB*, *ybhF*, *yadG*, and *yddA*). The X-axis indicates the target promoter probes, and the Y-axis shows the total count of differentially enriched proteins. Three categories are distinguished by color: low-abundance proteins; gray bars: Total differentially enriched proteins; red bars: Highly enriched proteins. All pull-down assays were performed with two independent biological replicates. The threshold criteria for identifying differentially bound proteins (log₂ fold-change cutoff and FDR-adjusted *p*-value) and replicate-to-replicate abundance variance are provided in section 2.2.4. Statistical comparisons between groups are described in Materials and Methods.

Distinct protein enrichment profiles were observed across the five promoter constructs. The *msbA*, *macB*, and *ybhF* promoters captured a broader repertoire of putative DNA-binding proteins, consistent with the potential for more elaborate transcriptional control circuits governing these systems. In contrast, relatively fewer interacting partners were recovered for *yadG* (305 proteins) and *yddA* (265 proteins), suggesting a more restricted repertoire of interacting partners and potentially simpler regulatory inputs (Figure 2 and Table 2). Collectively, these proteomic data identify a core set of promoter-interacting proteins, providing a basis for subsequent mechanistic investigation.

**Table 2:** DNA pull-down binding proteins for each promoter.

Target promoter	Total differentially enriched proteins	Highly enriched proteins	Low-abundance proteins
MsbA	623	578	45
MacB	499	466	33
YbhF	625	597	28
YadG	305	279	26
YddA	265	152	113

All differentially enriched proteins were screened by dual statistical thresholds described in Section 2.3: log₂(fold change) ≥ 1 (raw FC ≥ 2) and FDR-adjusted *p* < 0.05 via two-tailed unpaired Student’s *t*-test with Benjamini–Hochberg correction. Only proteins satisfying both criteria were classified as highly enriched proteins and counted in Table 2.

#### 3.2.2 Visualization analysis of differentially enriched proteins

**Figure 3:**
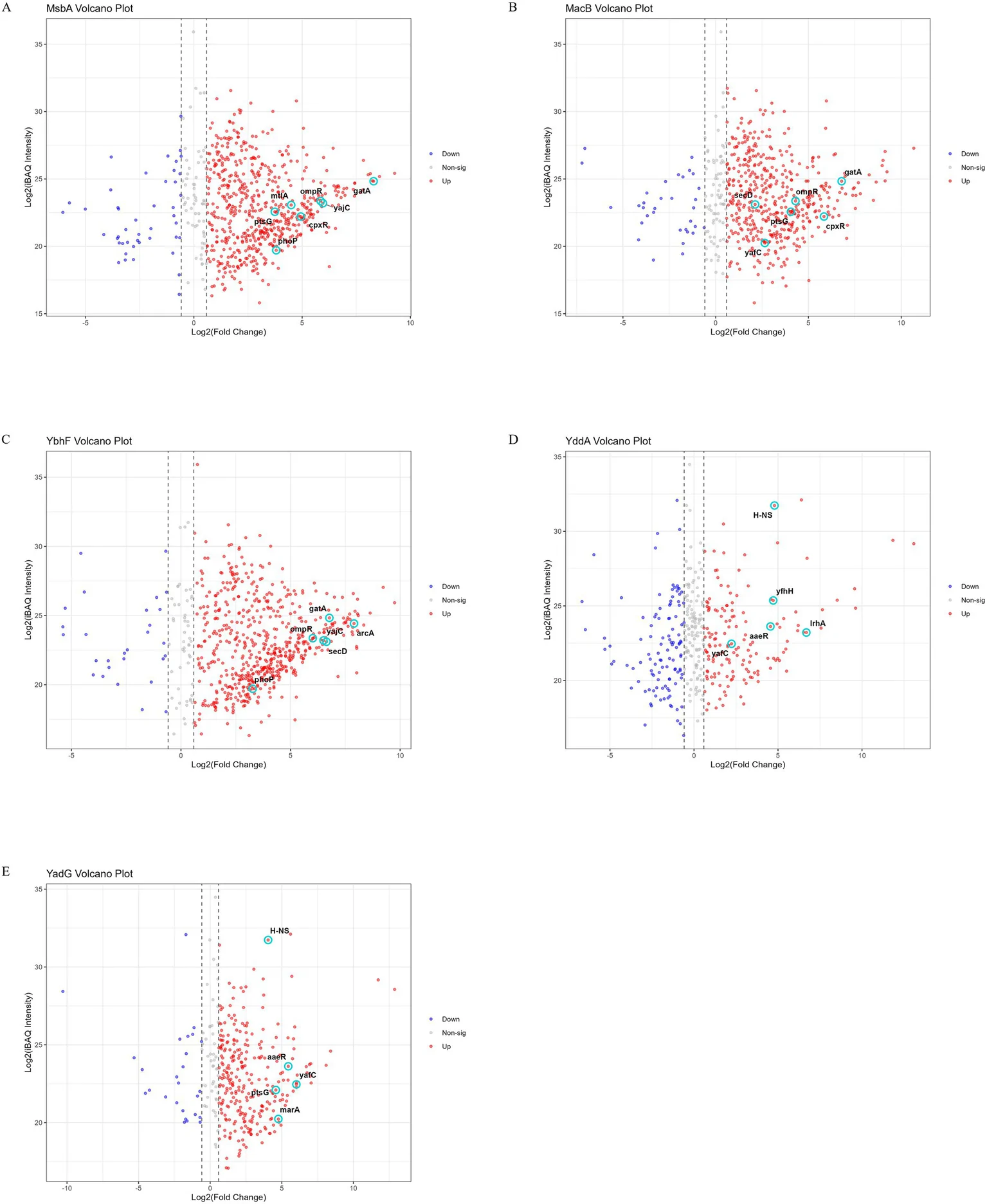
Volcano plot analysis of differentially enriched proteins captured by DNA pull-down assays targeting five ABC efflux pump promoters. The x-axis represents log₂(fold change; pull-down versus negative control), and the y-axis displays −log₁₀(*FDR*-adjusted *p* value). Two threshold lines are annotated on each panel: a vertical dashed line at log₂*FC* = 1 (fold change ≥ 2) and a horizontal dashed line at −log₁₀(0.05) (*FDR* = 0.05). Three protein categories are color-coded: red dots, significantly up-regulated proteins (log₂*FC* ≥ 1 and *FDR* < 0.05); gray dots, non-significant proteins; blue dots, significantly down-regulated proteins (log₂*FC* ≤ − 1 and *FDR* < 0.05). Candidate transcriptional regulators are individually labeled in each subgraph. **(A)** Volcano plot for the *msbA* promoter pull-down assay. Significantly enriched transcriptional regulators are highlighted. **(B)** Volcano plot for the *macB* promoter pull-down assay. Significantly enriched transcriptional regulators are highlighted. **(C)** Volcano plot for the *ybhF* promoter pull-down assay. Significantly enriched transcriptional regulators are highlighted. **(D)** Volcano plot for the *yddA* promoter pull-down assay. Significantly enriched transcriptional regulators are highlighted. **(E)** Volcano plot for the *yadG* promoter pull-down assay. Significantly enriched transcriptional regulators are highlighted.

Volcano plots were generated to visualize statistically significant binding proteins based on the thresholds described in section 2.3 (Figure 3). The horizontal axis represents log₂ (fold change, pull-down group vs. negative control), and the vertical axis denotes −log₁₀ (FDR-adjusted *p*-value). Two labeled threshold lines are drawn on every subgraph: vertical dashed line at log₂FC = 1 (FC = 2), horizontal dashed line at −log₁₀ (0.05) corresponding to FDR = 0.05. Three protein categories are distinguished by color: highly enriched proteins (strong binding to target promoter); blue dots = low-abundance proteins (weak binding signal relative to controls); gray dots = non-significant proteins failing either threshold. All five experimental groups exhibited marked enrichment of specific proteins compared with their matched negative controls.

For the MsbA, MacB, and YbhF groups, abundant protein hits fell within the upper-right quadrant (Figures 3A–C), suggesting these three promoters capture a broader range of interacting proteins under the assay conditions. CpxR was recovered in all three datasets, with the highest fold change observed in the MsbA group (FC = 30.57) (Figure 3A), consistent with a potential role in promoter binding. OmpR exhibited strong enrichment exclusively in the YbhF group (FC = 64.77) (Figure 3C), identifying OmpR as a candidate factor preferentially enriched at the *ybhF* promoter.

The YddA group presented a distinct enrichment profile. Significantly enriched factors, including H-NS (FC = 27.69) and LrhA (FC = 103.21), mostly fell within the medium-to-high abundance range (Figure 3D). The YadG group showed selective enrichment of multiple core transcriptional regulators (Figure 3E). The comparable distribution patterns shared by YbhF, MsbA, and MacB suggest these promoters may share overlapping binding patterns.

Key candidate proteins are annotated on each volcano plot (Figures 3A–E). OmpR, PhoP, and OmpC were co-enriched in the YbhF, MsbA, and MacB groups, consistent with a potential association with the OmpR/PhoP two-component signaling axis. In addition, both the MsbA and MacB groups captured abundant CpxR and GatA. The YddA group uniquely enriched LrhA, H-NS, AaeR, and YfhH, suggesting that these proteins represent candidate binding factors for the *yddA* promoter. The YadG group was distinguished by specific recovery of MarA, YafC, and H-NS. Together, these enrichment patterns suggest a candidate interaction network of DNA-binding proteins at these ABC transporter promoters. This putative network awaits follow-up experimental verification.

### 3.3 Screening and classification of candidate transcriptional regulators

Based on the statistically significant enrichment profiles identified above, we next classified candidate proteins with annotated transcriptional regulatory functions. We restricted downstream analyses to proteins annotated with transcriptional regulation (COG K), signal transduction (COG T), or DNA binding (GO:0003677), consistent with our screening objective. This functional filtering excluded abundant metabolic enzymes—the predominant source of background in bacterial lysates—including phosphotransferase system (PTS) proteins, which are discussed further below.

Differential enrichment was quantified via iBAQ intensity values. We applied a fold-change threshold of no less than 2; proteins satisfying this cutoff alongside positive enrichment signals were regarded as significantly enriched. Fourteen proteins fulfilled these criteria and carry annotated or predicted roles in chromatin organization or transcriptional control, making them candidate transcriptional regulators (Table 2). Distinct enrichment patterns were observed across the five promoter constructs (Table 3).

**Table 3:** Differential enrichment analysis of key transcriptional regulators.

Regulator	Category	MsbA FC	MacB FC	YbhF FC	YddA FC	YadG FC	Known function
OmpR	TCS	2.5	1.9	64.77	N. D.	N. D.	Osmotic stress, porin expression
PhoP	TCS	2.8	3.5	9.67	N. D.	N. D.	Mg^2+^ starvation, Pho regulon
CpxR	TCS	30.57	57.17	17.39	N. D.	N. D.	Envelope stress response
ArcA	Global	4.73	12.27	238.28	1	2.29	Redox/anaerobic regulation
MarA	Global	N. D.	N. D.	N. D.	N. D.	27.16	Multidrug resistance
SoxS	Global	3.1	N. D.	N. D.	N. D.	N. D.	Oxidative stress
H-NS	Nucleoid	1.8	N. D.	1.5	27.69	16.55	Gene silencing
GatA	PTS	315.12	110.54	109.39	0.28	2.35	Galactitol PTS
PtsG	PTS	13.34	16.61	13.74	1.28	24.03	Glucose PTS
MtlA	PTS	22.54	1.6	29.9	3.16	12.77	Mannitol PTS
OmpC	Outer membrane	3.17	1.53	6.87	0.5	12.94	Outer membrane porin
LrhA	HTH	N. D.	N. D.	N. D.	103.21	N. D.	HTH regulator
YafC	HTH	2.84	6.26	1.8	4.71	64.96	HTH regulator
AaeR	HTH	N. D.	2.45	0.87	23.44	43.89	Transcriptional activator

N. D. (not detected) indicates that the protein was not identified by mass spectrometry in the corresponding pull-down sample (iBAQ = 0).

For the *msbA*, *macB*, and *ybhF* promoters, TCS factors occupied the top ranks of enriched proteins based on fold change values. Notably, OmpR (FC = 64.77) and PhoP (FC = 9.67) were captured at the *ybhF* promoter, whereas CpxR (FC = 57.17) was identified at the *macB* promoter. These TCS proteins displayed promoter-specific enrichment profiles: CpxR and OmpR were undetectable at the *yddA* and *yadG* promoters, pointing to promoter-selective enrichment shaped by local DNA sequence features.

In contrast, the *yddA* promoter yielded abundant nucleoid-associated proteins and HTH-domain transcription factors, including H-NS (FC = 27.69), YfhH (FC = 26.31), AaeR (FC = 23.44), and LrhA (FC = 103.21), while ArcA showed negligible enrichment (FC = 1.0). This unique enrichment profile aligns with distinct putative regulatory mechanisms acting on *yddA*, which differ from the TCS-dominated signals recovered at the other four promoters.

The *yadG* promoter recruited a panel of global transcription factors, including MarA (FC = 27.16), AaeR (FC = 43.89), and Crr (FC = 14.97).

Recovered candidate factors fall into three functional classes: TCS signaling proteins (OmpR, PhoP, CpxR), global transcription factors (MarA, ArcA), and HTH-domain regulators (YfhH, AaeR, LrhA).

Beyond these candidate transcription factors, OmpC—an outer membrane porin whose abundance correlates with OmpR activity—was co-enriched alongside OmpR, implying potential physical coupling or functional linkage between inner-membrane ABC efflux machineries and outer-membrane permeability; this relationship requires dedicated experimental validation.

YafC, an HTH-containing protein, was markedly enriched at the *yadG* promoter (FC = 64.96), representing an additional candidate for follow-up functional characterization. PTS components were highly abundant in all pull-down samples with fold-change values exceeding 100, yet they were also readily detected in both negative control groups. Given their high cellular abundance, soluble state and negative surface charge, their recovery most likely arises from non-specific electrostatic interactions rather than sequence-specific DNA association, and they were therefore excluded from downstream regulator screening.

Promoter groups were defined by the enrichment profiles of functionally annotated TCS regulators (GO:0000160), rather than by total protein counts. TCS regulators were prominently enriched at the *msbA*, *macB*, and *ybhF* promoters, but remained largely undetectable at *yddA* and *yadG* (Table 3).

Having identified a set of candidate transcriptional regulators based on fold-change enrichment, we next performed systematic functional annotation of the full pull-down proteomic dataset to contextualize these candidates within broader biological pathways.

### 3.4 Quality control and functional annotation of the pull-down proteomic dataset

Mass spectrometry data quality was evaluated using standard QC metrics, including peptide length distribution, protein identification confidence, and trypsin digestion efficiency. All five promoter-targeted datasets met the predefined quality thresholds, supporting the robustness of downstream protein identifications.

To elucidate the potential functions of the pulled-down proteins, we performed systematic bioinformatics annotation. Although the dataset contained many metabolic enzymes common in bacterial lysates (background noise), functional enrichment analysis identified a distinct subset of proteins potentially involved in regulatory processes.

**Figure 4:**
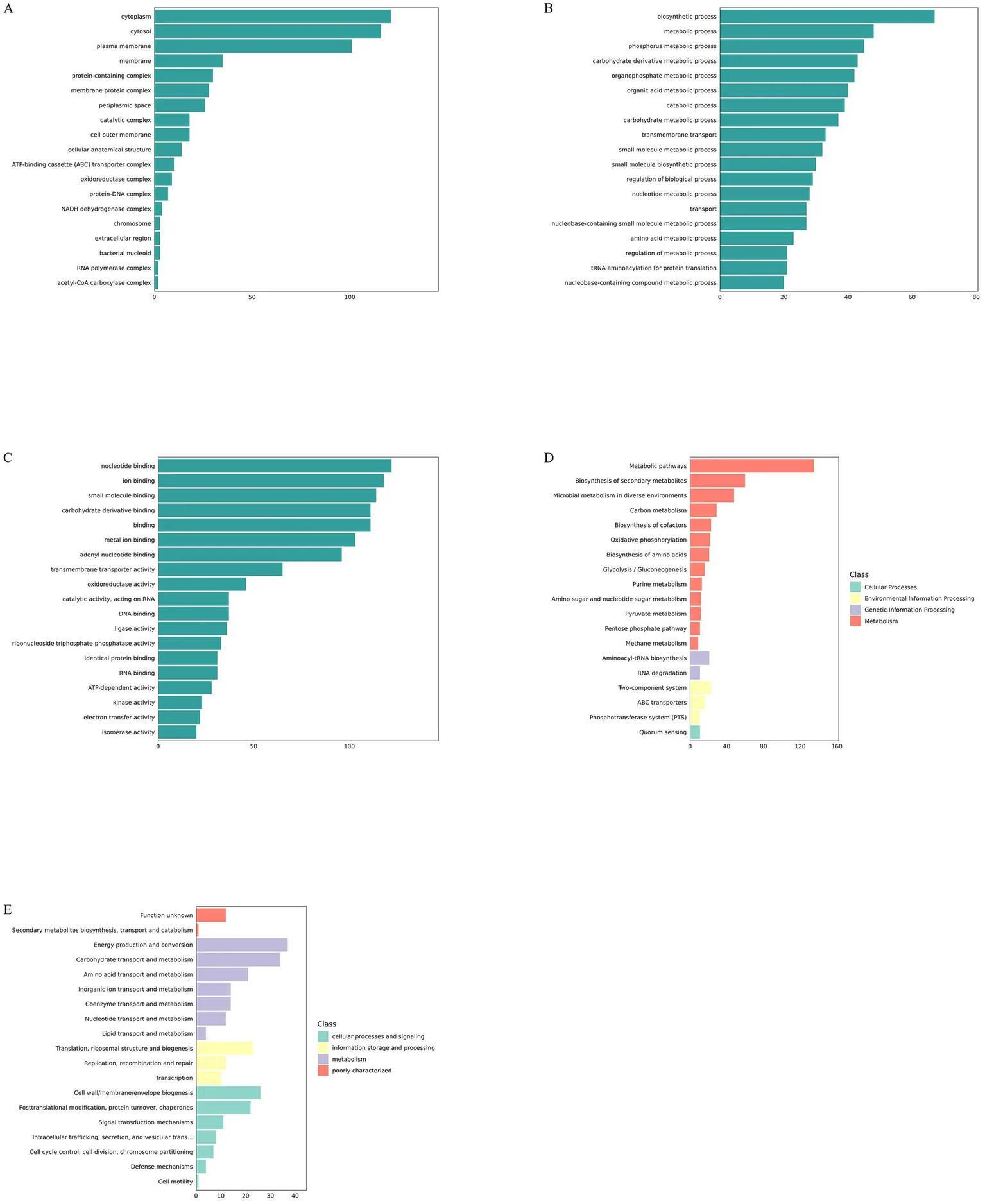
Functional enrichment classification and subcellular localization prediction of candidate interacting proteins captured by biotin-labeled *msbA* promoter probe via DNA pull-down assay. **(A)** Gene Ontology (GO) molecular function (MF) enrichment classification. The top enriched term “binding” refers to the general molecular activity of macromolecular/ligand binding; most proteins annotated under this term are transcription factors with DNA-binding domains, which target the *msbA* promoter DNA sequence to exert transcriptional regulatory functions. **(B)** GO biological process (BP) enrichment classification of captured proteins. **(C)** GO cellular component (CC) enrichment classification, reflecting the subcellular distribution of candidate regulatory proteins. **(D)** KEGG pathway enrichment analysis, showing the main metabolic and signal transduction pathways where these regulatory proteins participate. **(E)** Predicted subcellular localization distribution of all candidate proteins pulled down by *msbA* promoter probe.

We annotated the pulled-down proteins using standard GO and KEGG enrichment pipelines. Figure 3 shows the results for the MsbA pull-down; comparable enrichment patterns were obtained for MacB, YbhF, YddA, and YadG (Supplementary Figures S5–S8). Although metabolic enzymes accounted for most of the dataset, likely representing background binding, enrichment analysis identified a focused set of proteins linked to transport regulation and stress signaling (Figure 4).

GO functional annotation revealed significant enrichment for DNA-binding and nucleic acid–binding activities (MF), in addition to common metabolic functions (Figure 4A). Biological process (BP) analysis highlighted transcriptional regulation and cellular response to stimulus as the dominant categories (Figure 4B). This functional distribution is consistent with the presence of candidate transcriptional regulators within the pull-down protein set, although these annotations alone cannot confirm their actual regulatory roles *in vivo*.

COG classification revealed that while proteins assigned to “Amino acid transport and metabolism” constituted a major fraction of the captured cohort, hits annotated as “Transcription” and “Signal transduction mechanisms” were also identified (Figure 4C). This observation indicates the pull-down assay recovered candidate regulatory proteins together with abundant metabolic enzymes.

KEGG pathway analysis indicated overrepresentation of proteins annotated in ABC transporters, TCS, phosphotransferase systems (PTS), and bacterial secretion systems. Pathways related to biofilm formation were also over-represented (Figure 4D). The overrepresentation of TCS and PTS components among pull-down products aligns with a potential function of these signaling systems in tuning transcription of the ABC transporter genes examined, and this constitutes a testable hypothesis for future functional assays. This functional hypothesis remains to be validated through promoter–reporter assays and genetic mutant analysis.

Subcellular localization. The majority of candidate proteins localized to the cytoplasm and membrane (Figure 4E), matching the expected distribution of cytoplasmic regulators and membrane-bound transporters.

Although abundant metabolic proteins formed the primary background pool, functional classification recovered a subset of proteins annotated as TCS regulators, helix-turn-helix (HTH) transcription factors, and PTS components. Collectively, these functional annotations support the prioritization of the aforementioned candidates for subsequent functional characterization.

### 3.5 Conserved domain and structural feature analysis of ABC transporters

**Figure 5:**
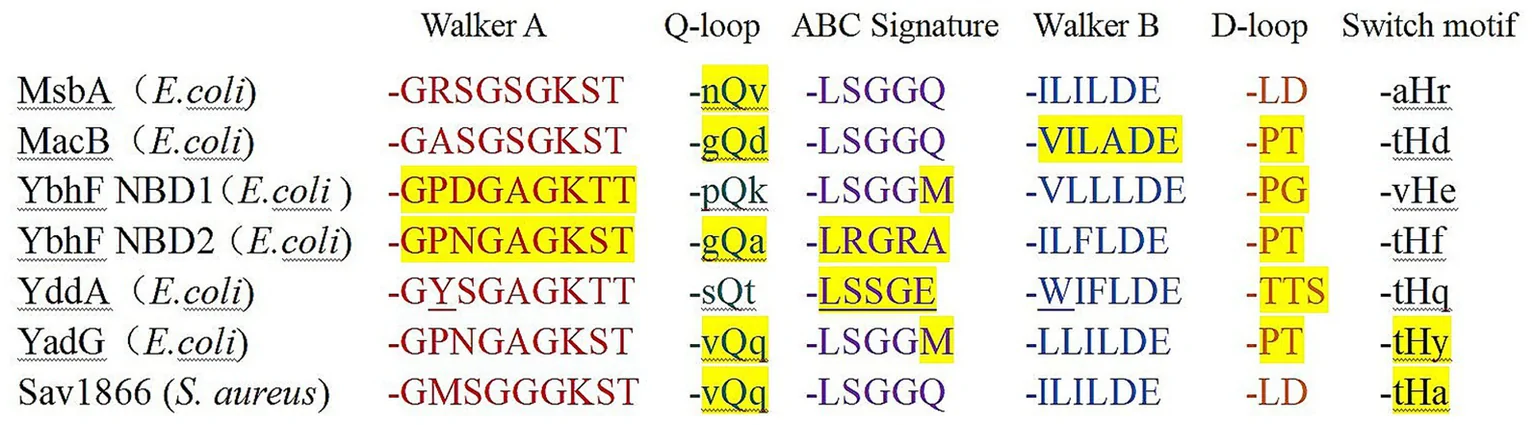
Multiple sequence alignment of conserved nucleotide-binding domain (NBD) sequences from ABC multidrug efflux transporters. The alignment includes sequences from *E. coli* [MsbA, MacB, YbhF (NBD1 and NBD2), YddA, and YadG] and *S. aureus* Sav1866, which serves as a structural reference. Six canonical ABC transporter functional motifs—Walker A, Q-loop, ABC signature, Walker B, D-loop, and Switch motif—are labeled on the alignment. The alignment was generated with Clustal Omega and visualized using Jalview.

Using NCBI CDD and SMART databases with the parameters described in section 2.5, we compared the conserved domains of five ABC efflux pumps from *E. coli* (MsbA, MacB, YbhF, YddA, YadG) against Sav1866 of *S. aureus* (Figure 5). All transporters except YddA display canonical ABC transporter structural features. MsbA and MacB are single-polypeptide fusion proteins containing one canonical NBD and one TMD, whereas YadG solely encodes a standalone NBD subunit. By contrast, the YbhF polypeptide harbors two tandem NBD domains (NBD1 and NBD2). Their core catalytic motifs, including Walker A, ABC signature and Walker B, show high sequence conservation relative to the classic Sav1866 template. Notably, all six functional motifs of YadG share nearly identical sequences with NBD1 of YbhF, verifying YadG as a typical canonical ABC NBD without atypical sequence alterations.

Among the five *E. coli* ABC transporters characterized here, YddA carries obvious sequence divergence at two catalytically critical NBD motifs according to CDD annotation. Most strikingly, its Walker A motif (GYSGAGKTT) carries an unusual tyrosine substitution at position 2, where all other examined ABC transporters contain non-aromatic residues such as Arg, Ala, Pro, or Met. Structural studies indicate that the flexibility of the phosphate-binding loop (P-loop) depends on small residues at this position; aromatic substitution may introduce steric hindrance that could impair ATP coordination (Rees et al., 2009). In addition, the ABC signature motif of YddA largely deviates from the canonical LSGGQ consensus and lacks the conserved diglycine core required to form the bipartite ATP-binding pocket at the NBD dimer interface. Moderate sequence variations were also detected within the Walker B and D-loop regions of YddA, while the Q-loop and H-switch motif still maintain their conserved core glutamine and histidine residues, respectively. These distinct structural features distinguish YddA from the other four ABC transporters examined. These in silico analyses provide only preliminary mechanistic clues; dedicated biochemical assays are required to determine whether these motif variations affect YddA-mediated multidrug efflux activity.

The *ybhFSR* locus forms a split-type operon with functional domains encoded by separate genes within a single transcriptional unit. Unlike MsbA, which functions as a homodimer of a single fusion polypeptide, the core functional domains of the *ybhFSR* operon are split across three independent open reading frames: *ybhF* encodes two NBDs, and *ybhS* and *ybhR* separately produce the TMD subunits.

Full amino acid sequences of all ABC transporters examined in this study, with manually annotated six core catalytic NBD motifs and matching NCBI reference accession numbers, are provided in Supplementary file S1.

### 3.6 Structural prediction of the YbhG-YbhF-TolC efflux complex assembly

Unlike canonical RND-type periplasmic adaptor proteins, which lack cytoplasmic extensions, analyses of topology predictions and multiple sequence alignments revealed that YbhG harbors an N-terminal transmembrane domain (Supplementary Figure S9), a structural trait lacking in canonical RND and MFS PAPs (Long et al., 2010; Hinchliffe et al., 2013). This feature is conserved among MacB-family adaptors and may facilitate transient cytoplasmic contacts with NBD-containing subunits such as YbhF during pump assembly (Greene et al., 2018; Santiago et al., 2017).

**Figure 6:**
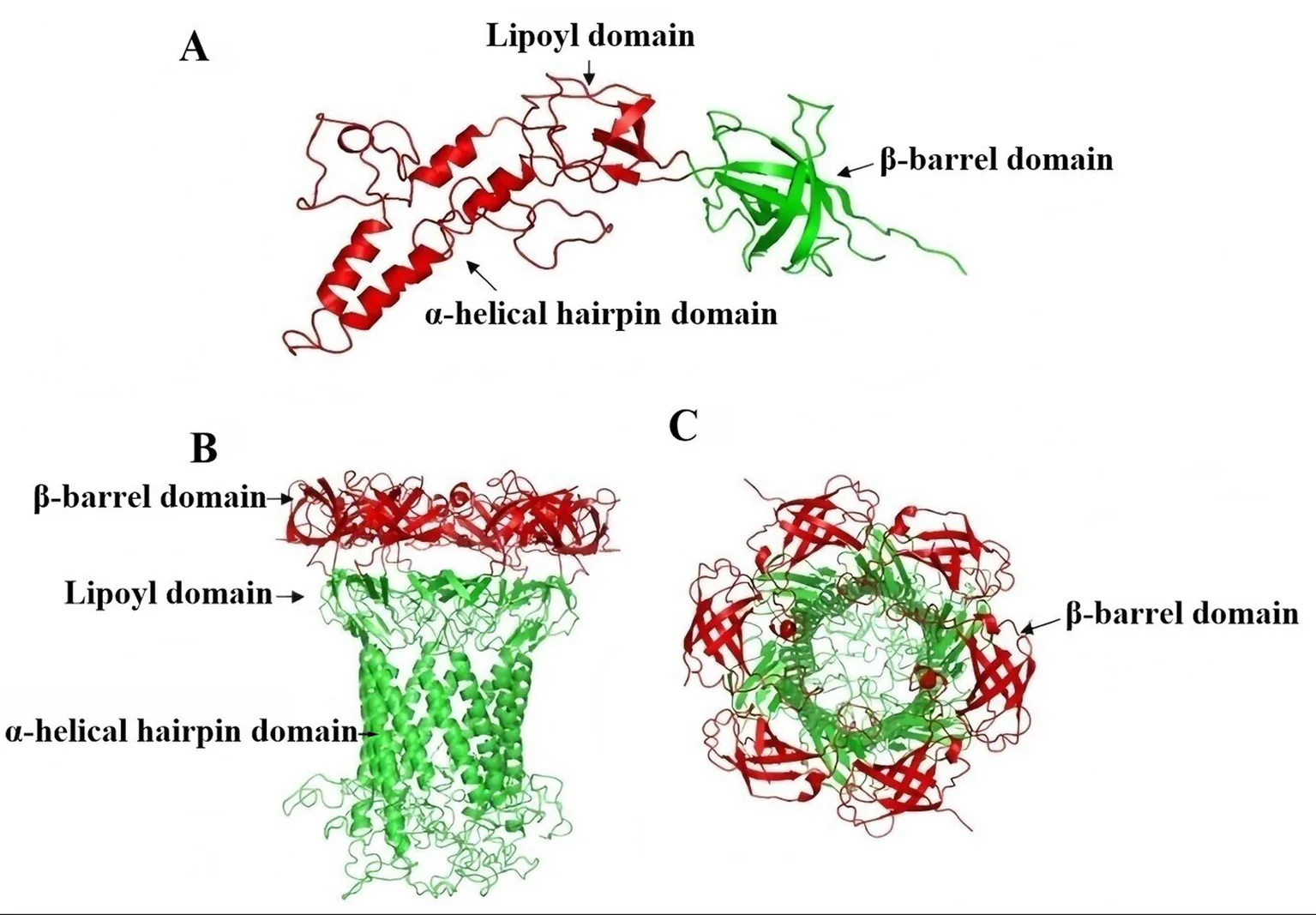
Three-dimensional structural cartoon representations of the *E. coli* YbhG protein monomer and its homohexameric assembly. **(A)** Cartoon model of a YbhG monomer. Distinct structural domains are color-coded: α-helical hairpin domain in red, lipoyl domain in light red, and β-barrel domain in green. Arrows indicate each independent structural module. **(B)** Lateral view of the YbhG homohexamer. Subunits are colored as in panel A to distinguish the α-helical hairpin, lipoyl, and β-barrel domains. **(C)** Cytoplasmic bottom view of the YbhG homohexamer, showing the circular arrangement of six β-barrel domains surrounding the central cavity. Structural models were visualized with PyMOL software.

Conserved domain analysis revealed that YbhG contains an N-terminal lipoyl domain, a central β-barrel domain, and a C-terminal *α*-helical hairpin domain (Figure 6). This three-domain architecture is characteristic of well-characterized periplasmic adaptor protein (PAP) family members, suggesting that YbhG possesses the core structural features typical of adaptor proteins. STRING network analysis further predicted tight functional coupling among YbhG, YbhF, YbhS, and YbhR, with combined confidence scores of 0.996, 0.993, and 0.992, respectively. These high-confidence interactions are consistent with their co-localization within a single operon in the *E. coli* genome, implying coordinated participation in a shared biological pathway.

**Figure 7:**
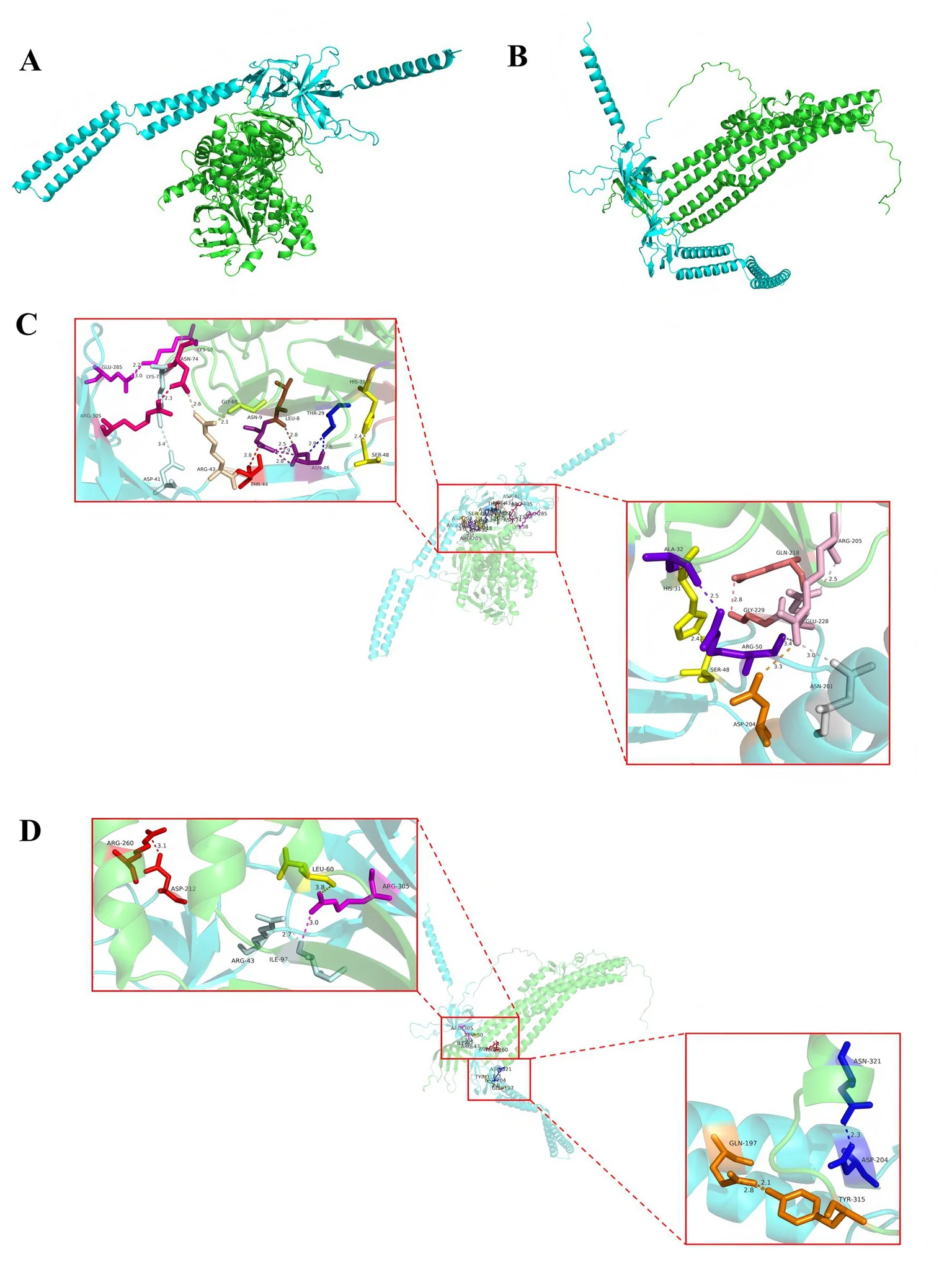
Predicted interaction models of the *E. coli* periplasmic adaptor YbhG with YbhF and TolC. All models were constructed by homology modeling followed by molecular docking using PDBePISA and visualized with PyMOL. **(A)** Cartoon representation of the predicted YbhG-YbhF heteromeric complex. **(B)** Cartoon representation of the predicted YbhG-TolC heteromeric complex. **(C)** Two-dimensional schematic of the YbhG-YbhF interface, highlighting hydrogen bonds and salt bridges with corresponding interatomic distances. **(D)** Two-dimensional schematic of the YbhG-TolC interface, highlighting hydrogen bonds and salt bridges with corresponding interatomic distances. Quantitative interface parameters, including numbers of hydrogen bonds and salt bridges, are provided in section 3.2.

Guided by these domain attributes, molecular docking simulations were performed to characterize interprotein binding interfaces. Docking outputs indicated that YbhF forms a compact, energetically favorable complex with YbhG. Their complementary binding interface was stabilized by 16 hydrogen bonds and five salt bridges, including key contacts such as a 2.85 Å hydrogen bond between YbhF Asn9 and YbhG Thr44, and a 2.18 Å salt bridge formed by YbhF Lys58 and YbhG Glu285. Moreover, the N-terminal region of YbhG was predicted to associate with the periplasmic domain of TolC, forming a putative complex supported by nine hydrogen bonds and four salt bridges, including key contacts such as YbhG Arg78–TolC Thr6 and YbhG Glu93–TolC Arg34 (Figure 7).

Collectively, these in silico predictions outline a plausible assembly mode for a tripartite efflux complex comprising YbhG, YbhF, and TolC. These computational models require experimental validation to confirm their physiological relevance. Mangu et al. (2026) illustrated that membrane-associated tripartite machineries rely on flexible, transient protein interfaces rather than rigid binding for reversible assembly. Consistent with this paradigm, the variable contact surfaces between YbhG and YbhF predicted here likely facilitate dynamic complex disassembly after substrate transport.

### 3.7 Orthogonal validation of the screening pipeline

To provide orthogonal validation of our screening pipeline, we conducted a parallel study (Feng et al., 2020) in which one representative candidate pair, MarA–*yadG*, was functionally characterized. Using EMSA, ChIP-qPCR, promoter-reporter fusions, and *marA* deletion analyses, we demonstrated that MarA binds directly to the *yadG* promoter and activates its transcription *in vivo* (Feng et al., 2020). This result supports the utility of our DNA pull-down approach for identifying functional transcriptional regulators. Systematic functional characterization of additional high-priority candidates—including CpxR, OmpR, and LrhA—is ongoing and will be reported separately.

## 4 Discussion

### 4.1 Distinct promoter-associated protein profiles of five ABC efflux systems

DNA pull-down coupled with LC–MS/MS revealed stark differences in enriched protein profiles across the five ABC transporter promoter probes. The *msbA, macB,* and *ybhF* fragments captured far more candidate DNA-associated proteins than *yad*G and *yddA* (Table 2; Figure 5), implying multilayered transcriptional networks control the former, while the latter are regulated by simpler regulatory circuits. This divergence fits with existing knowledge: while these ABC transporters share conserved nucleotide-binding domains, their regulatory strategies are tailored to genomic context and physiological roles (Davidson et al., 2008; Rees et al., 2009; Orelle et al., 2019).

Enriched transcriptional regulators also differed markedly. Promoters of *msbA*, *macB* and *ybhF* preferentially recruited TCS proteins, including CpxR, OmpR, PhoP, and ArcA (fold change 2.5–238.28; Table 3). CpxR has previously been implicated in *macB* regulation (Hu et al., 2022). The co-enrichment of multiple TCS regulators at these ABC promoters raises the possibility that cross-talk between TCS signaling and ABC efflux control may be far more pervasive than currently appreciated. By contrast, no TCS regulators exhibited significant enrichment in *yddA* or *yadG* pull-down assays. Instead, the *yddA* promoter specifically enriched the nucleoid-associated protein H-NS (FC = 27.69), together with HTH-family factors such as LrhA (FC = 103.21), AaeR and YfhH. Likewise, the *yadG* probe enriched H-NS (FC = 16.55), the global regulator MarA (FC = 27.16) and HTH-type YafC (FC = 64.96). H-NS functions as a broad transcriptional silencer for horizontally acquired loci, including numerous resistance genes (Dorman, 2004). Its consistent enrichment at *yddA* and *yadG* promoters suggests these systems are governed via silencing–derepression cycles rather than canonical TCS signaling. Our companion study independently demonstrates that MarA directly activates yadG transcription (Feng et al., 2020), supporting the interaction captured in this pull-down experiment. Collectively, these observations indicate ABC transporter transcription is tuned to distinct physiological demands: envelope stress-responsive pumps are coupled to rapid TCS cascades, whereas compact systems such as YddA and YadG are governed by nucleoid-associated and HTH transcriptional regulators.

Volcano plots confirmed the statistical robustness of these enrichment patterns (Figures 3A–E). PTS components and general metabolic enzymes were repeatedly recovered across all samples with high apparent fold changes, yet their signal intensities were comparable to those measured in negative controls. This artifact stems from the extremely high cellular abundance of these proteins: even weak non-specific interactions, including hydrophobic contacts, transient hydrogen bonding or passive adsorption onto streptavidin beads, can produce strong MS signals, a well-documented limitation of affinity proteomics (Trinkle-Mulcahy et al., 2008). To minimize such background, we adopted functional filtering alongside two independent negative controls. Metabolic enzymes without annotated DNA-binding activity were excluded irrespective of fold change; this approach is widely implemented to reduce false positives in DNA pull-down experiments (Butter and Vermeulen, 2010). Following filtering, TCS and HTH regulators were selectively enriched only in promoter pull-down samples, supporting interaction specificity and prioritizing these candidates for orthogonal validation. This regulatory dichotomy mirrors the structural variation among these efflux pumps, explored in subsequent sections.

Notably, among these candidate interactions, the regulatory connection between CpxR and *macB* has been previously validated (Hu et al., 2022). Transcriptome profiling by Barbosa and Levy (2000) further indicated that *yadG* expression is elevated upon constitutive MarA production, offering correlative evidence linking MarA to *yadG* regulation. In contrast, most protein-promoter associations identified here represent previously unreported regulatory interactions, including binding of multiple TCS regulators to the *msbA* and *ybhF* promoters, as well as coordinated occupancy by H-NS and diverse HTH factors at the *yddA* locus.

These findings extend current understanding of ABC efflux regulation. Beyond individually documented regulator-target pairs, we reveal two distinct global regulatory frameworks coordinating five related ABC transporters. This provides a testable working model describing how bacteria deploy separate signaling cascades to tune efflux function under distinct physiological stresses.

### 4.2 Structural diversity shapes the assembly modes of five ABC efflux pumps

The five systems differ markedly in their operon organization and structural assembly, yet they also share certain structural connections. MacB and YbhFSR may both function as tripartite efflux complexes involving periplasmic adaptor proteins and outer membrane channels. The NBD sequence of YadG shares high similarity with NBD1 of YbhF, suggesting a possible common evolutionary origin. YddAB exhibits a structural design distinct from the above systems, consisting of an inner-membrane and outer-membrane two-subunit assembly.

#### 4.2.1 Tripartite complexes: MacB and YbhFSR

MacB and YbhFSR may both belong to tripartite ABC efflux complexes that involve periplasmic adaptor proteins and outer membrane channels, though their internal organizations differ.

MacB is assembled from the inner-membrane protein MacB (containing NBD and TMD), the periplasmic adaptor protein MacA, and the outer membrane channel TolC (Greene et al., 2018). Its operon is organized as a *macA*-*macB*-*tolC* co-transcriptional unit. MacB forms a homodimer embedded in the inner membrane and transfers substrates to TolC via MacA, forming a continuous trans-envelope channel.

YbhFSR exhibits a more extreme split-type architecture. *ybhF* encodes two NBDs, while *ybhS* and *ybhR* encode the TMD subunits (Feng et al., 2020). YbhG may serve as the periplasmic adaptor protein for this system and belongs to the MacB family of PAPs together with MacA. MacB family PAPs (MacA and YbhG) possess a distinctive N-terminal cytoplasmic domain (Greene et al., 2018). MacA has a conserved N-terminal single transmembrane helix and a long flexible cytoplasmic tail (Greene et al., 2018). TMHMM topology prediction and sequence alignment of the N-terminal 35 amino acids of MacA and YbhG (38.1% identity, 52.4% similarity) suggested that YbhG may contain a homologous cytoplasmic extension. Its cytoplasmic tail is predicted to be rich in basic amino acid residues, which may facilitate electrostatic interactions with the acidic surface of the NBD domain. *In vitro* experiments on MacA demonstrated that truncation of its cytoplasmic tail completely abolished its ability to activate ATP hydrolysis by the MacB NBD, suggesting that direct physical contact between the cytoplasmic tail and the NBD may be required for energy coupling (Souabni et al., 2021; Tikhonova et al., 2007). YbhG possesses a similar cytoplasmic extension and may participate in energy coupling through a comparable mechanism.

Based on the structural features and sequence-based predictions described above, YbhG may serve as a bridge connecting the inner-membrane YbhFSR complex to the outer membrane TolC channel. Regarding the biological significance of the YbhG-YbhF interaction, the dynamic assembly model proposed by Santiago et al. (2017) offers a possible explanatory framework. In that model, the interaction between the periplasmic adaptor protein and the cytoplasmic NBD exists only transiently during the free assembly intermediate stage (Santiago et al., 2017). The YbhG-YbhF interaction may follow a similar dynamic pattern. We therefore speculate that the YbhG-YbhF interaction detected in this study may correspond to a transient assembly event prior to the formation of the mature complex. This interpretation does not conflict with the high-resolution structure of MacAB-TolC and provides clues for understanding the dynamic assembly process of ABC efflux pumps.

The assembly of MacB involves three components: MacB, MacA, and TolC. The assembly of YbhFSR may involve five components: YbhF, YbhS, YbhR, YbhG, and TolC. As a split-type system, YbhFSR has its NBD and TMD encoded by three separate genes, and its assembly process may be more complex, potentially requiring additional regulatory mechanisms to coordinate the expression levels of individual subunits. In the DNA pull-down assays, the promoters of both tripartite complexes enriched TCS regulators including CpxR, OmpR, PhoP, and ArcA, albeit with different enrichment intensities. The YbhF promoter captured more regulatory factors than the MacB promoter (Tables 2, 3).

#### 4.2.2 Inner-membrane two-subunit system: YadGH

YadGH is encoded by the yadG and yadH genes in a single operon. YadG is predicted to be an NBD, and YadH is predicted to be a TMD. Together they may form an inner-membrane two-subunit ABC transporter. Sequence alignment showed that the NBD of YadG shares high similarity with NBD1 of YbhF, suggesting a possible common evolutionary origin (Davidson et al., 2008; Higgins, 2001). However, no gene encoding a periplasmic adaptor protein or an outer membrane channel component was identified within the yadGH operon. Whether YadGH forms a tripartite complex with TolC remains uncertain, though this possibility appears limited. In the DNA pull-down assays, the YadG promoter did not enrich any TCS regulators. Instead, it mainly bound H-NS (FC = 16.55), MarA (FC = 27.16) and YafC (FC = 64.96) (Table 3). This regulatory profile differs markedly from that of YbhF.

#### 4.2.3 Inner-membrane–outer-membrane two-subunit system: YddAB

YddAB is encoded by the *yddA* and *yddB* genes in a single operon. YddA encodes a fusion polypeptide containing both NBD and TMD, while YddB encodes an outer membrane β-barrel protein. Together they may constitute an inner-membrane–outer-membrane transport unit.

As noted in section 3.5, YddA harbors atypical sequence signatures within its Walker A (GYSGAGKTT) and ABC signature (LSSGE) motifs, which differ markedly from the consensus sequences of canonical ABC transporters. These divergent motifs raise an important question: does YddA function via a conventional ATP-dependent transport mechanism, or utilize an alternative mode of energy coupling? If these amino acid substitutions compromise ATP binding or hydrolysis, YddA could constitute a functionally divergent ABC family member. It may rely on substrate-triggered conformational rearrangements or interactions with auxiliary protein factors to facilitate transport. On the other hand, these sequence variations may merely reflect evolutionary drift without diminishing catalytic capacity. Distinguishing between these scenarios requires dedicated biochemical characterization.

In the DNA pull-down assays, the YddA promoter did not enrich any TCS regulators. Instead, it mainly bound H-NS (FC = 27.69) and LrhA (FC = 103.21) (Table 3). LrhA is known to be involved in biofilm formation and stationary-phase adaptation (Lehnen et al., 2002). We therefore speculate that H-NS may mediate basal silencing of *yddAB*, while LrhA may participate in its transcriptional regulation under specific physiological conditions such as biofilm formation or stationary phase. This silencing–derepression regulatory mode has been described in several bacterial efflux pumps (Dorman, 2007), but its specific mechanism in YddAB awaits experimental validation.

#### 4.2.4 Summary

Based on the structural features of the five systems described above, their differences in operon organization and structural assembly are summarized in Table 4.

**Table 4:** Operon organization and structural features of the five ABC systems.

Class	System	Operon composition	Structural features
Tripartite complex (single-polypeptide inner-membrane protein)	MacB	*macB* + *macA*	NBD + TMD fusion + MacA + TolC
Tripartite complex (split type)	YbhFSR	*ybhF* + *ybhS/R* + *ybhG*	Dual NBD + TMD (separate) + YbhG (putative) + TolC
Inner-membrane two-subunit system	YadGH	*yadG* + *yadH*	NBD + TMD (separate), no PAP/TolC
Inner-membrane–outer-membrane two-subunit system	YddAB	*yddA* + *yddB*	NBD + TMD fusion + outer membrane β-barrel

MacB and YbhF may both be tripartite complexes, and their functions may both involve assembly across the inner membrane, periplasm and outer membrane. However, as a split-type system, YbhF has its NBD and TMD encoded by three separate genes, which may involve additional mechanisms to coordinate subunit expression. The bacterial two-hybrid assay detected a positive interaction signal between YbhG and YbhF, providing preliminary experimental evidence for the involvement of YbhG in the assembly of this system. YbhG, together with MacA, belongs to the MacB family of PAPs, and its N-terminal cytoplasmic tail may interact transiently with YbhF through a dynamic assembly mechanism similar to that described for MacA (Santiago et al., 2017). YadGH shares high sequence similarity with the YbhF system and may have a common evolutionary origin, but lacks the periplasmic adaptor protein and outer membrane channel components. YddAB exhibits a structural design distinct from the above systems. The structural classification outlined above provides a framework for future functional studies; however, the substrate specificity and efflux function of each system await experimental validation.

### 4.3 Functional stratification correlates with structural complexity

CpxR, OmpR, PhoP, and ArcA are well-characterized global transcriptional regulators whose roles in envelope stress responses, osmotic adaptation, and redox homeostasis have been extensively documented (Xu et al., 2014; Wu et al., 2021; Mao et al., 2025). However, direct binding of these regulators to the *ybhF*, *msbA*, *yddA*, and *yadG* promoters has not been systematically examined, except for one report linking CpxR to *macB* regulation (Hu et al., 2022). The parallel profiling of these established regulators across five ABC transporter promoters in this study therefore provides a comparative dataset that begins to address this gap.

In addition, our screening suggests several transcription factor–promoter interactions hitherto unreported in the context of ABC transporter regulation. Specifically, YafC was enriched at the *yadG* promoter (FC = 64.96); AaeR was enriched at the *macB* (FC = 2.45), *yddA* (FC = 23.44), and *yadG* (FC = 43.89) promoters; and YfhH was enriched at the *yddA* promoter. To our knowledge, none of these HTH-type regulators have previously been implicated in ABC transporter transcription. LrhA—a regulator known primarily for its roles in flagellar motility and biofilm formation—also showed enrichment at the *yddA* promoter (FC = 103.21), representing a previously unreported binding interaction.

Beyond these individual binding pairs, our data point to a possible structural–regulatory correlation. Multi-subunit tripartite ABC systems (MsbA, MacB, YbhF) showed preferential enrichment of envelope stress–responsive TCS regulators (CpxR, OmpR, PhoP, ArcA). In contrast, the compact bicistronic *yddA–yddB* operon was primarily associated with H-NS and HTH-type regulators (LrhA, YfhH, YafC, AaeR). This differential pattern has not been described in earlier studies and, if validated, may offer a useful framework for understanding ABC transporter regulation in *E. coli*.

Gahlot et al. (2023) demonstrated that chaperone-usher fimbria operons use divergent promoters to integrate environmental signals. Our ABC transporter screening reveals a similar architectural logic: despite sharing membrane-associated functions, these loci have evolved distinct composite promoters with non-overlapping transcription factor binding sites, mirroring the regulatory diversification reported for adhesive organelles.

### 4.4 CpxR as a candidate hub regulator in envelope stress and ABC pump control

Previous studies have demonstrated that Cpx-signaling can coordinate envelope remodeling and antimicrobial resistance phenotypes, supporting its broader role as a stress-responsive regulatory hub (Gahlot et al., 2024). While structural differences define their assembly modes, the next question is whether they share common transcriptional regulators. CpxR was among the most highly enriched regulatory proteins at the *msb*A, *macB*, and *ybhF* promoters in our pull-down assays. The CpxRA two-component system acts as a core pathway for Gram-negative bacteria to cope with envelope stress (Raivio, 2013). Our data suggest that CpxR may be involved in the transcriptional regulation of these ABC efflux pumps. This Cpx-mediated regulatory axis further connects envelope homeostasis with biofilm phenotypic remodeling (Gahlot et al., 2022).

Under external stress, CpxR likely functions as a key regulatory node. Our pull-down data imply that CpxR may influence the expression of multiple ABC pumps to strengthen bacterial defense. *msbA* expression rises to sustain LPS flipping (Bisht et al., 2024). *MacB* is induced to eliminate accumulated toxic substances. The YbhF system gets activated to balance ion homeostasis. This regulatory pattern is consistent with the clinical observation that outer membrane-targeted antimicrobials frequently trigger multidrug resistance (Olaitan et al., 2014). The CpxR/CpxA two-component system emerged as a candidate pathway from our *in vitro* binding data, but this observation requires functional validation (Dbeibo et al., 2018; Kwon and Lu, 2022).

### 4.5 OmpR, ArcA, and LrhA connect ABC pumps to microenvironmental adaptation

In addition to CpxR, OmpR, ArcA, and LrhA also exhibited promoter-specific enrichment in our DNA pull-down mass spectrometry data. OmpR was enriched at the *ybhF* promoter (FC = 64.77), whereas ArcA showed substantially higher enrichment at the same promoter (FC = 238.28). This strong signal may reflect specific protein–DNA interactions, but could also arise from non-specific background binding. Orthogonal validation, such as EMSAs, is required to distinguish between these possibilities.

The selective enrichment of OmpR and ArcA at the *ybhF* promoter is consistent with the possibility that these transcription factors associate with this DNA fragment *in vitro*. Given their documented roles in osmotic and redox signaling, we propose that OmpR and ArcA may modulate *ybhF* and *macB* transcription under hyperosmotic or anaerobic conditions. Direct testing of this hypothesis, however, would require intracellular or phenotypic experiments beyond the current dataset.

LrhA was also enriched at the *yddA* promoter, suggesting an association with this regulatory region. Notably, Cpx signaling and LrhA cooperatively control biofilm development, forming an adaptive network connecting ABC transporters to sessile growth (Gahlot et al., 2022). The functional significance of this interaction remains to be characterized.

Consistent with our earlier research (Feng et al., 2020), the YbhFSR complex is associated with cellular ion balance and may facilitate bacterial host colonization rather than acting primarily as an antibiotic efflux transporter. The enrichment of OmpR—an osmotic stress-responsive regulator—at the *ybhF* promoter offers preliminary proteomic evidence for this proposed model. Approximately 5% of the *E. coli* genome encodes ABC transporters that perform diverse biological functions independent of antimicrobial extrusion. As suggested by Piddock (2006), the mild antibiotic tolerance conferred by certain ABC pumps may represent secondary consequences of their primary roles in endogenous substrate transport.

### 4.6 Translational implications and future prospects

The revealed regulatory network provides preliminary theoretical hints; all intervention schemes remain unvalidated by drug screening and bacterial phenotype tests. Targeting shared regulatory hubs such as CpxR may allow simultaneous modulation of multiple ABC pump genes, although this remains a premise requiring experimental validation. Inhibitors designed to block subunit interaction interfaces of split-type YbhF structure can reduce off-target toxicity caused by NBD targeting (Ambudkar et al., 1999). Interference with derepression mechanisms helps restrain H-NS-silenced pumps like YddA, which benefits biofilm-associated infection treatment. Resistance risk can be assessed according to local infection conditions. Environment-sensing intervention serves as a promising approach to relieve efflux-mediated bacterial tolerance.

## 5 Research limitations and future directions

DNA pull-down experiments were performed in-house, whereas LC–MS/MS acquisition was conducted by BioGenes GmbH. All experimental workflows and bioinformatic analysis pipelines were designed and implemented by our group.

### 5.1 Limitations of the *in vitro* DNA pull-down system

This study employed an in vitro DNA pull-down approach to capture protein-DNA complexes from bacterial whole-cell lysates. Detected interactions therefore represent binding events under lysis buffer conditions and reflect a snapshot of the post-translational modification landscape of extracted proteins. Regulators that depend on intracellular cofactors, stimulus-induced modifications, or the native cellular microenvironment for promoter engagement may not be recovered in this assay. All protein–promoter associations reported here lack direct *in vivo* validation. Orthogonal assays—such as EMSAs, ChIP-qPCR, and promoter–reporter constructs—will be required to confirm physical binding and to determine whether each regulator activates or represses transcription. The regulatory roles proposed for CpxR, OmpR, ArcA, and LrhA are therefore best considered testable hypotheses at this stage.

### 5.2 Limitations of *in silico* structural prediction

The conserved domain analyses and protein–protein docking models of YbhF, YbhG, and TolC presented here are computational predictions. No site-directed mutagenesis, co-crystallization, or *in vitro* binding assays were undertaken to validate the predicted tripartite assembly interface. The proposed YbhG-YbhF-TolC assembly model therefore remains a computational construct requiring biochemical validation.

### 5.3 Absence of phenotypic and *in vivo* functional verification

This study did not include antibiotic susceptibility testing, biofilm assays, or animal infection models. Direct experimental evidence linking the identified transcription factors to osmotic adaptation, anaerobic responses, biofilm formation, or host-site-specific resistance is therefore not available from the current dataset. These proposed correlations warrant independent functional validation.

### 5.4 Translational therapeutic hypotheses remain preliminary

The therapeutic strategies discussed here—such as targeted modulation of the CpxR regulatory node or disruption of YbhF subunit interfaces—remain speculative without empirical validation. This study did not include compound screening, inhibitor profiling, or *in vivo* efficacy studies. These translational avenues therefore require independent follow-up and lie beyond the scope of the current dataset.

Of note, functional validation of the MarA-*yadG* interaction is reported in a companion manuscript currently under review (Feng et al., 2020), providing independent support for the reliability of our screening pipeline. Systematic mechanistic validation of key candidates—including CpxR, OmpR, PhoP, and LrhA—is ongoing and will be reported separately. The present study provides a proteomic and structural framework; comprehensive functional dissection of these regulatory networks will require further experimental investigation.

## 6 Conclusion

In this work, we integrated DNA pull-down with LC–MS/MS, bioinformatic annotation, conserved domain analysis and molecular docking to characterize the regulatory profiles of five representative ABC efflux pumps in *E. coli*.

Our data point to a dichotomy: multi-subunit ABC efflux systems (MsbA, MacB, YbhF) predominantly associate with two-component system regulators, whereas the YddA and YadG mainly interact with H-NS and HTH-type transcription factors. This regulatory divergence correlates with structural complexity; split-operon architectures appear to be coupled to distinct transcriptional regulatory networks.

All identified protein-promoter interactions were captured in cell-free *in vitro* assays and require orthogonal validation. The regulatory models proposed here should therefore be viewed as testable hypotheses rather than definitive conclusions.

## Data Availability

The data presented in the study are deposited in the iProX and ProteomeXchange repositories, accession number IPX0018707000 and PXD082106.

## Supplementary material

Supplementary Figure S1 Agarose gel validation of the biotinylated *macB* promoter fragment and silver-stained SDS-PAGE analysis of pull-down eluates. Left panel: Agarose gel showing the biotinylated *macB* promoter probe. Lane M, DNA marker; control, negative control PCR; experiment, biotinylated probe PCR product. Right panel: Silver-stained SDS-PAGE of pull-down samples, normalized by BCA quantification for equal loading. Lane M, protein molecular weight marker; Control, eluates from negative control pull-down; Experiment, eluates from probe-bound pull-down; Input, whole-cell soluble lysate.

Supplementary Figure S2 Agarose gel validation of the biotinylated *ybhF* promoter fragment and silver-stained SDS-PAGE analysis of pull-down eluates. Left panel: Agarose gel showing the biotinylated *ybhF* promoter probe. Lane M, DNA marker; control, negative control PCR; experiment, biotinylated probe PCR product. Right panel: Silver-stained SDS-PAGE of pull-down samples, normalized by BCA quantification for equal loading. Lane M, protein molecular weight marker; Control, eluates from negative control pull-down; Experiment, eluates from probe-bound pull-down; Input, whole-cell soluble lysate.

Supplementary Figure S3 Agarose gel validation of the biotinylated *yddA* promoter fragment and silver-stained SDS-PAGE analysis of pull-down eluates. Left panel: Agarose gel showing the biotinylated *yddA* promoter probe. Lane M, DNA marker; control, negative control PCR; experiment, biotinylated probe PCR product. Right panel: Silver-stained SDS-PAGE of pull-down samples, normalized by BCA quantification for equal loading. Lane M, protein molecular weight marker; Control, eluates from negative control pull-down; Experiment, eluates from probe-bound pull-down; Input, whole-cell soluble lysate.

Supplementary Figure S4 Agarose gel validation of the biotinylated *yadG* promoter fragment and silver-stained SDS-PAGE analysis of pull-down eluates. Left panel: Agarose gel showing the biotinylated *yadG* promoter probe. Lane M, DNA marker; control, negative control PCR; experiment, biotinylated probe PCR product. Right panel: Silver-stained SDS-PAGE of pull-down samples, normalized by BCA quantification for equal loading. Lane M, protein molecular weight marker; Control, eluates from negative control pull-down; Experiment, eluates from probe-bound pull-down; Input, whole-cell soluble lysate.

Supplementary Figure S5 Functional enrichment and subcellular localization analysis of proteins captured by the *macB* promoter probe. **(A)** Gene Ontology (GO) molecular function (MF) classification. **(B)** GO biological process (BP) classification. **(C)** GO cellular component (CC) classification. **(D)** KEGG pathway enrichment analysis. **(E)** Subcellular localization prediction of captured proteins.

Supplementary Figure S6 Functional enrichment and subcellular localization analysis of proteins captured by the *ybhF* promoter probe. **(A)** Gene Ontology (GO) molecular function (MF) classification. **(B)** GO biological process (BP) classification. **(C)** GO cellular component (CC) classification. **(D)** KEGG pathway enrichment analysis. **(E)** Subcellular localization prediction of captured proteins.

Supplementary Figure S7 Functional enrichment and subcellular localization analysis of proteins captured by the *yddA* promoter probe. **(A)** Gene Ontology (GO) molecular function (MF) classification. **(B)** GO biological process (BP) classification. **(C)** GO cellular component (CC) classification. **(D)** KEGG pathway enrichment analysis. **(E)** Subcellular localization prediction of captured proteins.

Supplementary Figure S8 Functional enrichment and subcellular localization analysis of proteins captured by the *yadG* promoter probe. **(A)** Gene Ontology (GO) molecular function (MF) classification. **(B)** GO biological process (BP) classification. **(C)** GO cellular component (CC) classification. **(D)** KEGG pathway enrichment analysis. **(E)** Subcellular localization prediction of captured proteins.

Supplementary Figure S9 TMHMM topology prediction for YbhG. Probability profile indicating a single N-terminal transmembrane helix. The C-terminal portion of YbhG is predicted to be located within the periplasm. Lines denote posterior probabilities for transmembrane segments (purple), cytoplasmic localization (inside, light blue), and periplasmic localization (outside, orange).
