# Supplementary material for: From drug export to envelope fitness: characterizing the regulatory landscape of selective Escherichia coli ABC efflux pumps
Source: Front Microbiol. 2026 Sep 4;17:1889688. doi: 10.3389/fmicb.2026.1889688 (PMC13585988; doi:10.3389/fmicb.2026.1889688)
Supplement: Supplementary file 1 [file Data_Sheet_1.PDF]

MsbA ATP-binding lipopolysaccharide transport protein [*Escherichia coli* str. K-12 substr. MG1655]

NCBI Reference Sequence: NP_415434.1

>NP_415434.1 ATP-binding lipopolysaccharide transport protein [*Escherichia coli* str. K-12 substr. MG1655]

MHNDKDLSTWQTFRRLWPTIAPFKAGLIVAGVALILNAASDTFMLSLLKPLLDDGFGKTDRSVLVWMPLV

VIGLMILRGITSYVSSYCISWVSGKVVMTMRRRLFGHMMGMPVSFFDKQSTGTLLSRITYDSEQVASSSS

GALITVVREGASIIGLFIMMFYYSWQLSIILIVLAPIVSIAIRVVSKRFRNISKNMQNTMGQVTTSAEQM

LKGHKEVLIFGGQEVETKRFDKVSNRMRLQGMKMVSASSISDPIIQLIASLALAFVLYAASFPSVMDSLT

AGTITVVFSSMIALMRPLKSLTNVNAQFQRGMAACQTLFTILDSEQEKDEGKRVIERATGDVEFRNVTFT

YPGRDVPALRNINLKIPAGKTVALVGRSGSGKST(Walker A)IASLITRFYDIDEGEILMDGHDLREYTLASLRNQVA(Q-loop)

LVSQNVHLFNDTVANNIAYARTEQYSREQIEEAARMAYAMDFINKMDNGLDTVIGENGVLLSGGQ(ABC Signature)RQRIAIARALLRDSPILILDE(Walker B)ATSALD(D-loop)TESERAIQAALDELQKNRTSLVIAHR(Switch motif)L

STIEKADEIVVVEDGVIVERGTHNDLLEHRGVYAQLHKMQFGQ

MacB ABC-type tripartite efflux pump ATP binding/membrane subunit [*Escherichia coli* str. K-12 substr. MG1655]

NCBI Reference Sequence: NP_415400.1 GenPept Identical Proteins Graphics

>NP_415400.1 ABC-type tripartite efflux pump ATP binding/membrane subunit [*Escherichia coli* str. K-12 substr. MG1655]

MTPLLELKDIRRSYPAGDEQVEVLKGISLDIYAGEMVAIVGASGSGKST(Walker A)LMNILGCLDKATSGTYRVAGQ

D(Q-loop)VATLDADALAQLRREHFGFIFQRYHLLSHLTAEQNVEVPAVYAGLERKQRLLRAQELLQRLGLEDRTEY

YPAQLSGGQ(ABC Signature)QQRVSIARALMNGGQVILADE(Walker B)PTGA(D-loop)LDSHSGEEVMAILHQLRDRGHTVII

VTHD(Switch motif)PQVAAQAERVIEIRDGEIVRNPPAIEKVNVTGGTEPVVNTVSGWRQFVSGFNEALTMAWRALA

ANKMRTLLTMLGIIIGIASVVSIVVVGDAAKQMVLADIRSIGTNTIDVYPGKDFGDDDPQYQQALKYDDLIAIQKQPWVASATP
AVSQNLRLRYNNVDVAASANGVSGDYFNVYGMTFSEGNTFNQEQLNGRAQVVVLDSNTRRQLFPHKADVVGEVILVGNMPA
RVIGVAEEKQSMFGSSKVLRVWLPYSTMSGRVMGQSWLNSITVRVKEGFDSAEAEQQLTRLLSLRHGKKDFFTWNMDGVLKT
VEKTTRTLQLFLTLVAVISLVVGGIGVMNIMLVSVTERTREIGIRMAVGARASDVLQQFLIEAVLVCLVGGALGITLSLLIAFTLQ
LFLPGWEIGFSPLALLLAFLCSTVTGILFGWLPARNAARLDPVDALARE

YbhF-NBD1 ABC exporter ATP binding subunit YbhF [*Escherichia coli* str. K-12 substr. MG1655]

NCBI Reference Sequence: NP_415315.4

>NP_415315.4 ABC exporter ATP binding subunit YbhF [*Escherichia coli* str. K-12 substr. MG1655]

MNDAVITLNGLEKRFPGMDKPAVAPLDCTIHAGYVTGLVGPDGAGKTT(Walker A)LMRMLAGLLKPDSGSATVIGFD

PIKNDGALHAVLGYMPQKF(Q-loop)GLYEDLTVMENLNLYADLRSVTGEARKQTFARLLEFTSLGPFTGRLAGKLS

GGM(ABC Signature)KQKLGLACTLVGEPKVLLLDE(Walker B)PGVG(D-loop)

VDPISRRELWQMVHE(Switch motif)LAGEGMLILWSTSYLDEAEQC

RDVLLMNEGELLYQGEPKALTQTMAGRSFLMTSPHEGNRKLLQRALKLPQVSDGMIQGKSVRLILKKEATPDDIRH

ADGMP

YbhF-NBD2 ABC exporter ATP binding subunit YbhF [*Escherichia coli* str. K-12 substr. MG1655]

NCBI Reference Sequence: NP_415315.4

>NP_415315.4 ABC exporter ATP binding subunit YbhF [*Escherichia coli* str. K-12 substr. MG1655]

EININETTPRFEDAFIDLLGGAGTSESPLGAILHTVEGTPGETVIEAKELTKKFGDFAATDHVNF

AVKRGEIFGLLGPNGAGKST(Walker A)TFKMMCGLLVPTSGQA(Q-loop)LVLGMDLKESSGKARQ

HLGYMAQKFSLYGNLTVEQNLRFFSGVYGLRGRA(ABC Signature)QNEKISRMSEAFGLKSIASHATDELPLGFKQRLALACSLMHEPDILFLDE(Walker B)PTSG(D-loop)VDPLTRREFWLHINSMVEKGVTVMVTTHF(Switch motif)MDEAEYCDRIGLVY

RGKLIASGTPDDLKAQSANDEQPDPTMEQAFIQLIHDWDKEHSNE

YddA ABC transporter family protein YddA [*Escherichia coli* str. K-12 substr. MG1655]

NCBI Reference Sequence: NP_416013.1

>NP_416013.1 ABC transporter family protein YddA [*Escherichia coli* str. K-12 substr. MG1655]

MITIPITLRMLIAKYLCLLKPFWLRKNNKTSVLLIIIILAMILGVVKIQVWLNDWNNDFFNALSQKETDK

LWQLVLWFPALLGIFVLISVNKTWLIKLLTIRWREWLTDYYLNRWFADKNYYFTQIYGEHKNTDNPDQRI

AEDILLLISKTLSLSFGFIQSLSMLITFTVILWESAGTLSFTVGGTEWNIQGYMVYTVVLIVIGGTLFTH

KVGKRIRPLNVEKQRSEATFRTNLVQHNKQAELIALSNAESLQRQELSDNFHTIKENWHRLMNRQRWLDY

WQNIYSRSLSVLPYFLLLPQFISGQINLGGLMKSRQAFMLVSNNLSWFIYKYDELAELAAVIDRLYEFHQ

LTEQRPTNKPKNCQHAVQVADASIRTPDNKIILENLNFHVSPGKWLLLKGYSGAGKTT(Walker A)LLKTLSHCWPWF

KGDISSPADSWYVSQT(Q-loop)PLIKTGLLKEIICKALPLPVDDKSLSEVLHQVGLGKLAARIHDHDRWGDILSSG

E(ABC Signature)KQRIALARLILRRPKWIFLDE(Walker B)TTSH(D-loop)LEEQEAIRLLR

LVREKLPTSGVIMVTHQ(Switch motif)PGVWNLADDICDISAVL

YadG putative ABC transporter ATP-binding protein YadG [*Escherichia coli* str. K-12 substr. MG1655]

NCBI Reference Sequence: NP_414669.1

>NP_414669.1 putative ABC transporter ATP-binding protein YadG [*Escherichia coli* str. K-12 substr. MG1655]

MTIALELQQLKKTYPGGVQALRGIDLQVEAGDFYALLGPNGAGKST(Walker A)TIGIISSLVNKTSGRVSVFGYDLE

KDVVNAKRQLGLVPQEFNFNPFETVQQI(Q-loop)VVNQAGYYGVERKEAYIRSEKYLKQLDLWGKRNERARMLSGG

M(ABC Signature)KRRLMIARALMHEPKLLILDE(Walker B)PTAG(D-loop)VDIELRRSMWGFLKDLNDKGTTIILTTHY(Switch motif)LEEAEMLCRNIGIIQHGELVENTSMKALLAKLKSETFILDLAPKSPLPKLDGYQYRLVDTATLEVEVLREQGINS

VFTQLSEQGIQVLSMRNKANRLEELFVSLVNEKQGDRA

SAV1866 family putative multidrug efflux ABC transporter [*Staphylococcus aureus*]

GenBank: AZL91854.1

>AZL91854.1 SAV1866 family putative multidrug efflux ABC transporter [*Staphylococcus aureus*]

MIKRYLQFVKPYKYRIFATIIVGIIKFGIPMLIPLLIKYAIDGVINNHALTTDEKVHHLTIAIGIALFIF

VIVRPPIEFIRQYLAQWTSNKILYDIRKKLYNHLQALSARFYANNQVGQVISRVINDVEQTKDFILTGLM

NIWLDCITIIIALSIMFFLDVKLTLAALFIFPFYILTVYVFFGRLRKLTRERSQALAEVQGFLHERVQGI

SVVKSFAIEDNEAKNFDKKNTNFLTRALKHTRWNAYSFAAINTVTDIGPIIVIGVGAYLAISGSITVGTL

AAFVGYLELLFGPLRRLVASFTTLTQSFASMDRVFQLIDEDYDIKNGVGAQPIEIKQGRIDIDHVSFQYN

DNEAPILKDINLSIEKGETVAFVGMSGGGKST(Walker A)LINLIPRFYDVTSGQILIDGHNIKDFLTGSLRNQIGLV

QQD(Q-loop)NILFSDTVKENILLGRPTATDEEVVEAAKMANAHDFIMNLPQGYDTEVGERGVKLSGGQ(ABC Signature)KQRLSIARIFLNNPPILILDE(Walker B)ATSALD(D-loop)LESESIIQEALDVLSKDR

TTLIVAHRLSTITHA(Switch motif)DKIVVIENGHIVETGTHRELIAKQGAYEHLYSIQNL
